# Tools for assessing quality and risk of bias in Mendelian randomization studies: a systematic review

**DOI:** 10.1093/ije/dyac149

**Published:** 2022-07-28

**Authors:** Francesca Spiga, Mark Gibson, Sarah Dawson, Kate Tilling, George Davey Smith, Marcus R Munafò, Julian P T Higgins

**Affiliations:** Population Health Sciences, Bristol Medical School, University of Bristol, Bristol, UK; Medical Research Council Integrative Epidemiology Unit, University of Bristol, Bristol, UK; Medical Research Council Integrative Epidemiology Unit, University of Bristol, Bristol, UK; School of Psychological Science, University of Bristol, Bristol, UK; Population Health Sciences, Bristol Medical School, University of Bristol, Bristol, UK; Population Health Sciences, Bristol Medical School, University of Bristol, Bristol, UK; Medical Research Council Integrative Epidemiology Unit, University of Bristol, Bristol, UK; Population Health Sciences, Bristol Medical School, University of Bristol, Bristol, UK; Medical Research Council Integrative Epidemiology Unit, University of Bristol, Bristol, UK; Medical Research Council Integrative Epidemiology Unit, University of Bristol, Bristol, UK; School of Psychological Science, University of Bristol, Bristol, UK; Population Health Sciences, Bristol Medical School, University of Bristol, Bristol, UK; Medical Research Council Integrative Epidemiology Unit, University of Bristol, Bristol, UK

**Keywords:** Mendelian randomization, genetic instrument, bias, tool, guideline, risk-of-bias assessment

## Abstract

**Background:**

The use of Mendelian randomization (MR) in epidemiology has increased considerably in recent years, with a subsequent increase in systematic reviews of MR studies. We conducted a systematic review of tools designed for assessing risk of bias and/or quality of evidence in MR studies and a review of systematic reviews of MR studies.

**Methods:**

We systematically searched MEDLINE, Embase, the Web of Science, preprints servers and Google Scholar for articles containing tools for assessing, conducting and/or reporting MR studies. We also searched for systematic reviews and protocols of systematic reviews of MR studies. From eligible articles we collected data on tool characteristics and content, as well as details of narrative description of bias assessment.

**Results:**

Our searches retrieved 2464 records to screen, from which 14 tools, 35 systematic reviews and 38 protocols were included in our review. Seven tools were designed for assessing risk of bias/quality of evidence in MR studies and evaluation of their content revealed that all seven tools addressed the three core assumptions of instrumental variable analysis, violation of which can potentially introduce bias in MR analysis estimates.

**Conclusion:**

We present an overview of tools and methods to assess risk of bias/quality of evidence in MR analysis. Issues commonly addressed relate to the three standard assumptions of instrumental variables analyses, the choice of genetic instrument(s) and features of the population(s) from which the data are collected (particularly in two-sample MR), in addition to more traditional non-MR-specific epidemiological biases. The identified tools should be tested and validated for general use before recommendations can be made on their widespread use. Our findings should raise awareness about the importance of bias related to MR analysis and provide information that is useful for assessment of MR studies in the context of systematic reviews.

Key Messages

- Mendelian randomization (MR) analyses are potentially a powerful approach to making causal inferences in observational studies, but they are built on important assumptions and are not immune to bias.
- We identified 14 tools for the conduct, evaluation and/or reporting of MR studies. We focused on the evaluation tools and most of these contained items addressing validity of the three core instrumental variables assumptions.
- We also examined systematic reviews of MR studies and protocols for systematic reviews. Only a small proportion of the reviews conducted a risk-of-bias assessment although most included a narrative description of MR-related bias and limitations. Most of the protocols planned to conduct an assessment, although fewer than half of them planned methods specifically for MR studies.
- The tools we identified should provide a useful source of information on what bias/limitations reviewers should be aware of when reading or systematically reviewing MR studies.

## Introduction

Mendelian randomization (MR) is an analytic approach used to make causal inference in observational studies.^1^ In MR analysis, genetic variants are generally used as instrumental variables (genetic instruments) to estimate the causal effect of a modifiable trait (the causal factor or ‘exposure’) on another trait (the factor or condition that the exposure is hypothesized to influence or ‘outcome’).^2^ Causal inference using MR analysis is based on the notion that genetic variants are randomly inherited from parents to offspring in a way that is comparable to participants being randomly allocated to each experimental group in a randomized–controlled trial (RCT).^3^ In a within-sibship analysis randomization is almost exact^4^ and MR was introduced through this hypothetical approach,^1^ but until recently large-scale data were not available to conduct such analyses and the approximate randomization in population-level data (adjusted for potential population stratification) has been the main approach.^3^ Thus, the key advantage of using a MR approach is the potential to reduce bias due to residual confounding and reverse causation, which are often limitations in other types of observational studies.^1^^,^^5^ MR was introduced as a way of strengthening causal inference regarding the effects of modifiable exposures studied in conventional observational epidemiological studies.^1^^,^^6^

As for instrumental variables analyses in general, the validity of an estimate from a MR analysis relies on the genetic instrument satisfying three core assumptions:^1^ the genetic instrument must be associated with the exposure (IV1-relevance),^2^ there are no unmeasured confounders of the genetic instrument–outcome association (IV2-independence) and^3^ the genetic instrument–outcome association must be mediated entirely via the exposure (IV3-exclusion restriction). Additional assumptions, which are variations of a fourth IV assumption (IV4),^2^^,^^7^ may be required for some inferences. Versions of these include (i) the association of the genetic instrument and the exposure and the effect of the exposure on the outcome are the same for all participants in the sample (homogeneity); (ii) the genetic instrument does not modify the effect of the exposure on the outcome within levels of the exposure and for all levels of the exposure (no effect modification); (iii) the direction of the effect of the exposure on the outcome is the same for all participants in the sample (monotonicity).^8^ Finally, to consider that the findings inform intervention strategies it must be assumed that the differences in an exposure induced by the genetic instrument will produce the same downstream effects on health outcomes as differences in the exposure produced by environmental influences (gene–environment equivalence assumption).^2^^,^^9^ The validity of two-sample MR studies, in which different samples are used to estimate the genetic instrument–exposure and genetic instrument–outcome associations, relies on additional assumptions that the samples are independent (i.e. do not overlap): the samples are from the same underlying population (e.g. same age range, same ancestry)^10^ (thus with the genetic variants being equally distributed in the two populations)^11^ and the genetic variants are harmonized (i.e. they are in the same direction in the two samples).^10^

Some of the specific biases that have been articulated in relation to MR studies include biases emerging from the genetic instrument (e.g. weak instrument bias,^12^ bias due to horizontal pleiotropy,^13^ bias due to linkage disequilibrium,^5^ bias due to developmental compensation)^1^ and biases related to the population from which the data are collected (e.g. bias due to population stratification, assortative mating, dynastic effect and parent of origin effect,^1^^,^^14^^,^^15^ bias due to sample overlap in two-sample MR).^16^ Using weak instruments in MR analysis creates a problem in relation to IV1 and can lead to estimates biased towards the confounded exposure–outcome association (in one-sample MR) or towards the null (in two-sample MR). Failure to adjust for population structure and familial effects can introduce confounding in a way that is similar to lack of randomization in a RCT and relates to IV2.^14^ Horizontal pleiotropy leads to violation of IV3. Some problems can lead to violation of more than one IV assumption; e.g. linkage disequilibrium can introduce both horizontal pleiotropy (IV3)^17^ and confounding (IV2).^5^ In addition, biased estimates can arise from other more general types of bias, including measurement/classification biases, selection biases (including those due to missing data and to collider bias) and reporting biases.

Since the initial detailed exposition of MR in epidemiology in 2003,^1^ its use has increased very considerably and with this has come a parallel increase in systematic reviews of MR studies. One important component of a systematic review (and meta-analysis) is the evaluation of the quality of evidence reported in each study included. This is increasingly achieved by assessing risk of bias through a structured framework. Although numerous tools for risk-of-bias assessment in studies of interventions have been developed for both RCTs^18^ and non-randomized studies of intervention,^19^ and are widely used, there is no widely agreed tool for assessing MR studies.

In this systematic review we sought to identify and examine structured frameworks used to assess risk of bias (or quality more generally) in MR studies. Specifically, we undertook a comprehensive and objective review of tools for the systematic evaluation of MR studies; identified and summarized tools for assessing the conduct and/or reporting of MR studies to examine what bias-related features they covered; and undertook an examination of how risk of bias in MR studies has been assessed in systematic reviews to date.

## Methods

### Eligibility criteria

For the review of existing tools, we sought structured guidelines, checklists and other tools aimed at comprehensive evaluation of the conduct, evaluation and/or reporting of MR studies or structured guidance through the steps of conducting or reporting an MR study. For the review of systematic reviews, we examined articles describing systematic approaches to collating and summarizing MR studies within a field or more generally. We considered a systematic review any article in which the authors (i) undertook a bibliographic database search (e.g. in MEDLINE and/or other databases); and (ii) provided a table describing each of the included studies. We included full reports (e.g. full-text articles) and protocols, but not conference abstracts (unless an associated full-text report could be identified). We regarded any article in which genetic variants have been described or used as instrumental variables as relevant to our review.

### Searches

We performed systematic electronic searches in (i) MEDLINE (Ovid), Embase (Ovid) and the Web of Science (from inception to 30 June 2021) for published peer-reviewed articles and (ii) bioRxiv and medRxiv for preprint articles (last search 1 July 2021). We implemented specific searches to identify articles describing tools (Search 1), systematic reviews (Search 2) and protocols for systematic reviews (Search 3). To identify systematic reviews, we also searched Epistemonikos and for information on ongoing reviews we searched PROSPERO and Open Science Framework (OSF) Registries (last search 1 July 2021). To identify additional articles and protocols (missed from the bibliographic database searches), we searched Google Scholar, examined references of included studies and performed forward citation searches (Google Scholar) to identify articles citing included studies. Details of search strategies are reported in Supplementary data (available as Supplementary data at *IJE* online).

### Study selection

Search results were managed using EndNote 20 and Excel. Titles and abstracts were screened by one review author (F.S.) using Rayyan app (www.rayyan.ai). The full text of selected studies was retrieved and assessed for eligibility and inclusion in the review. Full-text screening was performed independently by two review authors (F.S. and M.G.) and disagreements between the two reviewers were resolved through discussion. Any structured tool identified from the review of systematic reviews was incorporated into the review of tools.

### Data extraction

An extraction form was used to extract the data from the articles selected for inclusion. For each sub-review, a pilot data extraction was performed and a finalized data extraction form was compiled. From each article, the following general information was extracted by one review author (F.S.): first author(s) name and year of publication, type of report (full-text article or conference abstract), type of article (e.g. tool, systematic review, protocol of systematic review) and complete reference. In addition, information specific to the sub-reviews was extracted as follows:

Review of tools: number of tools within the article, purpose of the tool (i.e. conducting, evaluating or reporting), structure of the tool (e.g. guide, dictionary, checklist) and for the evaluating tools only, specific objectives of the article, other tools used as template, number of domains and items (or questions) and specific content of each item within each tool. We extracted information only about tools designed specifically for MR studies.

Review of systematic reviews: review topic, whether only MR studies were included, number of included MR and non-MR studies, whether a systematic assessment of risk of bias was undertaken (or proposed if a protocol) and, if applicable, whether a structured tool was used, what biases were addressed, how biases were addressed, if a narrative description of MR-specific bias was reported and what biases were narratively addressed. We also evaluated whether a systematic assessment of the quality of evidence supporting a causal effect reported by individual MR studies was undertaken and, if applicable, what approaches were used.

### Data analysis and reporting

We report our findings using structured summary tables and narrative descriptions. For the tools identified in the first sub-review that were aimed at the evaluation of an MR study, we tabulate the items addressed by the different tools. Where an item contained multiple questions, we separate these and tabulate each question as a single item. We mapped items across tools to examine how similar biases were addressed by different evaluating tools and to convey how many of the tools addressed each bias. Specifically, we classified each item into a broad bias/topic domain and then we assigned each item to a specific bias/topic within that domain and determined the numbers of items allocated to each bias domain and to specific MR bias/topic. For the systematic reviews, we tabulate the methods of risk-of-bias and/or quality-of-evidence assessment in MR studies and the MR-relevant bias addressed either by the method of assessment used or within a narrative description. For protocols of systematic reviews, we tabulate the proposed methods of assessment of risk of bias/quality of evidence in MR studies. Data extraction, narrative synthesis and tabulations were performed by one reviewer (F.S.).

## Results

### Tools for the conduct, evaluation and reporting of MR studies

**Figure 1:**
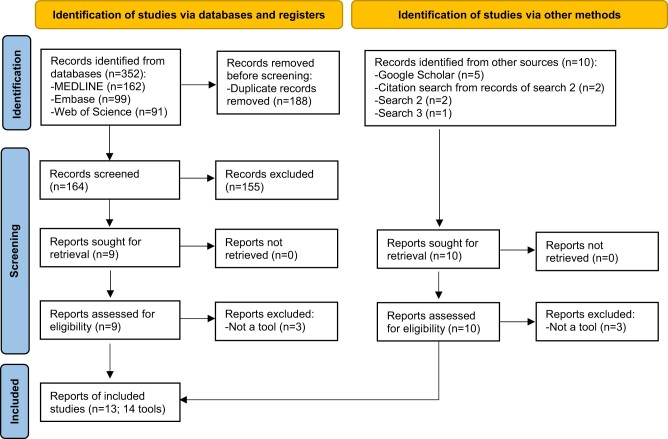
Flow diagram of identification, screening and inclusion of articles containing tools for the conduct, evaluation and reporting of Mendelian randomization studies

In total, 363 records were identified from the searches (352 from database searches and 11 from other searches) of which 19 were retrieved for full-text screening. The inclusion criteria were met by 13 articles (reporting 14 tools) that are included in this review. A flow diagram of the identification, screening and inclusion of articles is shown in Figure 1*.*Of the 13 included articles, 6 were identified from searches of electronic databases of peer-reviewed articles and 4 from searches of preprints archives and Google Scholar, 2 from cited references, 2 from searches of systematic reviews (Search 2) and 1 from searches of protocols of systematic reviews (Search 3). A list of all the included tools is reported in Supplementary Table S1 (available as Supplementary data at *IJE* online) and the six studies that did not meet the criteria for inclusion are listed in Supplementary data (available as Supplementary data at *IJE* online).

Of the 14 tools included, 8 tools were designed for single use in a specific systematic review (7 reviews and 1 protocol) and 6 tools were proposed for future use for the conduct, evaluation and/or reporting of MR studies in general or within the context of a systematic review. Of the 14 identified tools, 8 tools had a single purpose, of which 4 were aimed at the conduct of MR studies, 3 were aimed at the reporting of MR studies and 1 was aimed at the evaluation of MR studies. The remaining six tools had two purposes: evaluation and reporting of MR studies.

Details of the seven tools designed (or used) for evaluation of MR studies are reported in Table 1. Of these, Burgess,^20^ Davies,^21^ Grau-Perez^22^ and Treur^31^ were structured by domains and items, whereas Kuźma,^24^ LS Lee^26^ and Mamluk^28^ were structured by items only. The number of domains within the first four evaluating tools ranged from 5 to 9, with a median of 6 and a total of 26 domains across the tools. The number of items in the evaluating tools ranged from 5 to 28, with a median of 19 and a total of 121 items across all the tools.

**Table 1:** Details of tools designed/used for assessing risk of bias/evaluating quality in Mendelian randomization studies

Author	Objectives of the article	Tool used as template/reference to other tools or articles relevant to MR	*N* of domains	*N* of items or questions
Burgess 2020^20^	To provide guidelines for performing MR investigations. To provide advice on which analyses to perform in a MR investigation		9	22
Davies 2018^21^	To provide explanations of core concepts and recent developments in MR methods		6	19
Grau-Perez 2019^22^	To conduct a systematic review of MR studies evaluating the causal role of environmentally responsive DNAm changes on the development of health states	Boef *et al.*^23^	6	28
Kuźma 2018^24^	To conduct a systematic review of MR studies investigating causal relationships between risk factors and global cognitive function or dementia	Q-Genie^25^	–	11
Lee 2020^26^	To perform an updated systematic review and meta-analysis of MR that will provide further insight into the causative factors of dementia	Davies *et al*.^21^ Grover *et al.*^27^ Burgess *et al.*^20^	–	11
Mamluk 2020^28^	To conduct a systematic review of human studies that used experimental data or alternative analytical methods to determine the causal effects of maternal alcohol consumption in pregnancy on offspring outcomes at birth and later in life	Glymour *et al.*^29^ Lawlor *et al.*^15^ Taylor *et al.*^30^	–	5
Treur 2021^31^	To review evidence from studies that applied MR to assess causal effects between poor mental health and substance use	STROBE-MR^32^^,^^33^	5	25

DNAm, DNA methylation; MR, Mendelian randomization.

We conducted a thorough analysis of the structure and content of the evaluating tools by classifying each item into a bias/topic domain and then we assigned each item to a specific bias/topic. We found that of the 121 items among all evaluating tools, 81 items were designed to evaluate risk of bias in MR studies and 44 items were designed to address other aspects of the MR analysis (4 items were designed to address both evaluation of risk of bias and other aspects of MR analysis). Of the 81 items designed to evaluate MR studies, 77 addressed only one bias and 4 addressed multiple biases.

Details of the biases addressed by each evaluating tool are reported in Table 2. Of the 81 items addressing bias, 32 related to the three core IV assumptions. Ten items in 7 tools addressed bias related to the relevance assumption (IV1), 8 items in 6 tools addressed bias related to the independence assumption (IV2) and 14 items in 7 tools addressed bias related to the exclusion restriction assumption (IV3). In addition, 11 items in 4 tools addressed bias related to the selection of the genetic instrument and 14 items in 6 tools addressed bias related to the selection of the population(s) or sample(s). Five items in 4 tools addressed bias related to sensitivity analysis, 19 items in 3 tools addressed bias related to measurement errors and misclassification, 2 items in 1 tool addressed bias due to missing data, 4 items in 3 tools addressed bias due to other types of confounding and 2 items in 1 tool addressed other sources of bias. We provide details of the 44 items addressing other aspects of the MR analysis, including items addressing the reporting of MR analysis, in Supplementary Table S2 (available as Supplementary data at *IJE* online). Among these, we found that two items in one tool addressed clinical implications of the MR results; three items in three tools addressed the choice of data set(s); four items in three tools addressed the genetic instrument; six items in two tools addressed the interpretation of the MR analysis results; five items in three tools addressed the MR rationale; six items in three tools addressed the MR results; four items in three tools addressed precision of the results; two items in one tool addressed the selection of the population(s) or sample(s); and seven items in four tools addressed the statistical analysis.

**Table 2:** Details of specific Mendelian randomization bias and limitation addressed by items or questions within each assessing tool

Bias (or topic) domain	Specific bias or topic addressed by tool	Burgess 2020	Davies 2018	Grau-Perez 2019	Kuźma 2018	Lee 2020	Mamluk 2020	Treur 2021	Total items
IV1-Relevance	Choice of variants	Yes							1
	Weak instrument bias		Yes	Yes	Yes	Yes	Yes	Yes	9
IV2-Independence	Choice of variants	Yes							1
	Confounding		Yes	Yes	Yes	Yes^a^	Yes^b^	Yes	7
	Population stratification						Yes		1
IV3-Exclusion restriction	Choice of variants	Yes							1
	Horizontal pleiotropy	Yes	Yes	Yes	Yes	Yes	Yes	Yes	13
Genetic instrument	Choice of variants	Yes							3
	Construction of genetic score							Yes	1
	Variants harmonization	Yes	Yes			Yes		Yes	4
	Linkage disequilibrium	Yes	Yes			Yes			3
Population/sample	Samples overlap^a^	Yes	Yes			Yes		Yes	5
	Population heterogeneity^c^	Yes	Yes	Yes		Yes	Yes	Yes	6
	Choice of controls			Yes					1
	Selection bias			Yes					1
Sensitivity analysis	Evidence of robustness	Yes	Yes			Yes		Yes	5
Measurement error	Exposure measurement error/misclassification			Yes	Yes			Yes	13
	Outcome measurement error/misclassification			Yes	Yes			Yes	6
Missing data	Missing data			Yes					2
Other confounding	Non-MR-specific confounding			Yes^d^	Yes		Yes^e^		4
Other sources of bias	Traditional epidemiologic biases (i.e. non-MR-specific)				Yes^f^				2

a Confounding of the genetic instrument–outcome association.

b Confounding of the genetic instrument–exposure association and of the genetic instrument–outcome association.

c In two-sample MR analysis.

d Confounding factors for the exposure–outcome or mediator–outcome association.

e Included confounders in the IV analysis.

f Survival and diagnostic bias. IV1, instrumental variable assumption 1; IV2, instrumental variable assumption 2; IV3, instrumental variable assumption 3; MR, Mendelian randomization.

In addition to the evaluating tools, we identified three tools aimed at reporting and four tools aimed at conducting MR studies. All seven tools contained items addressing bias in MR analysis and details of the content of the items is reported in Supplementary Table S3 (available as Supplementary data at *IJE* online). The number of domains ranged from 3 to 6 in the reporting tools and from 5 to 10 in the conducting tools; the number of items ranged from 7 to 61 in the reporting tools and from 18 to 26 in the conducting tools. Among the reporting tools, all three tools contained items addressing the three IV core assumptions; Boef^23^ contained items addressing linkage disequilibrium and canalization; Davey Smith^32^ contained items addressing homogeneity and sample overlap (in two-sample MR); Lor^34^ contained items addressing linkage disequilibrium and heteroscedasticity. Among the conducting tools, Burgess,^20^ Grover^27^ and Lawlor^35^ contained items addressing the three IV core assumptions and variant harmonization; in addition, Burgess^20^ contained one item addressing the homogeneity assumptions and Grover^27^ and Lawlor^35^ contained items addressing sample overlap; Swerdlow^36^ contained items addressing linkage disequilibrium and horizontal pleiotropy.

### Systematic reviews of MR studies

#### Completed reviews

**Figure 2:**
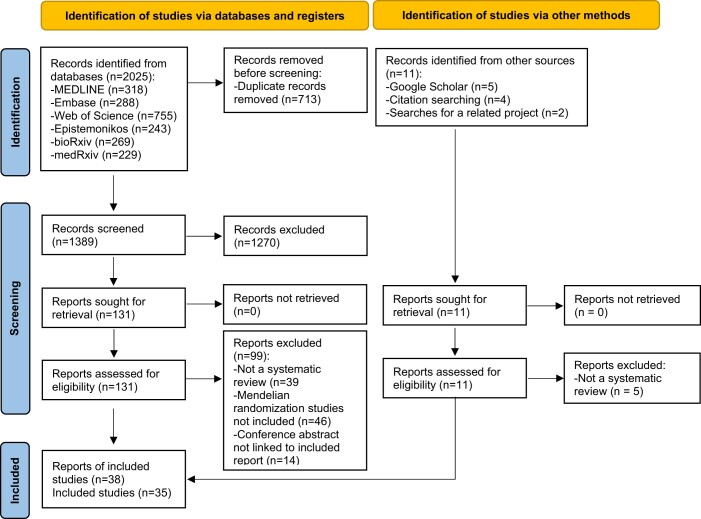
Flow diagram of identification, screening and inclusion of articles containing systematic reviews (and meta-analysis) of Mendelian randomization studies

A total of 2036 records were identified from Search 2 (for systematic reviews) (2025 from database searches and 11 from other searches) of which 143 were retrieved for full-text screening and the inclusion criteria were met by 38 articles (35 full-text articles and 3 conference abstracts linked to included articles) reporting 35 reviews that are included in this synthesis. A flow diagram of identification, screening and inclusion of studies is shown in Figure 2. A list of included reviews is reported in Table 3 and the 104 studies that did not meet the criteria for inclusion are listed in Supplementary data (available as Supplementary data at *IJE* online).

**Table 3:** List of included systematic reviews reporting one or more Mendelian randomization studies

Study ID	Type of article	Topic of the review	Were only MR studies included?	*N* of MR studies/*N* of non-MR studies	Risk-of-bias assessment in individual MR studies? If Yes, was a structured tool used?	Name and/or description of risk-of-bias assessment method	Evidence of causal effect assessment in individual MR studies? If Yes, was a structured method used?	Description of evidence of causal effect assessment method	Narrative description of MR-specific bias
Abbasi 2015^37^	Systematic review and MR analysis	MR studies of biomarkers and T2D	Yes	28/0	No	N/A	No	N/A	Yes
Abbasi 2016^38^	Systematic review	Biomarkers and T2D	No	17/122	No	N/A	No	N/A	Yes
Belbasis 2020^39^	Umbrella review	Risk factors and peripheral biomarkers for schizophrenia and other psychotic disorders	No	5/36	No	N/A	No	N/A	No
Belbasis 2018^40^	Umbrella review	Risk factors of multiple sclerosis	No	6/9	No	N/A	No	N/A	Yes
Bellou 2018^41^	Umbrella review	Environmental risk factors and biomarkers for T2D	No	22/86	No	N/A	No	N/A	Yes
Bergmans 2021^42^	Systematic review	Comorbid depression and T2D	No	4/12	No	N/A	No	N/A	Yes
Bochud 2010^43^	Literature review on MR methods, applications and limitations	MR studies	Yes	38/0	No	N/A	Yes/No	Strength of genetic variant	Yes
Boef 2015^a^,^23^	Systematic review	Methodology used in MR analysis	Yes	179/0	No	N/A	No	N/A	Yes
Carnegie 2020^44^	Literature review on MR methods, applications and limitations and systematic review of MR studies	MR in Nutritional psychiatry	Yes	26/0	No	N/A	No	N/A	Yes
Cheng 2020^45^	Systematic review and meta-analysis	Puberty timing and T2D and/or impaired glucose tolerance	No	1/27	Yes, Yes	Newcastle-Ottawa Scale^69^	No	N/A	No
Diemer 2021^46^	Systematic review	Prenatal environment and offspring outcomes	Yes	43/0	No	N/A	No	N/A	Yes
Firth 2020^47^	Umbrella review	Modifiable health behaviours and major mental disorders	No	12/32	No	N/A	Yes/No	Statistical analysis results, use of sensitivity analysis and test for bidirectional effects	Yes
Frayling 2018^48^	Systematic review	MR studies of T2D, coronary artery disease and hypertension	Yes	16/0	No	N/A	No	N/A	Yes
Grau-Perez 2019^a^,^22^	Systematic review	MR studies of environmentally responsive DNAm changes and the development of health states	Yes	15/0	Yes, Yes	Self-developed tool	No	N/A	Yes
Hu 2019^49^	Systematic review	MR studies of atherosclerotic cardiovascular disease	Yes	58/0	No	N/A	No	N/A	Yes
Kei 2018^50^	Systematic review	MR studies of serum uric acid levels and cardiovascular and renal disease risk	Yes	16/0	No	N/A	No	N/A	Yes
Kim 2020^51^	Umbrella review of systematic reviews and meta-analyses	Adiposity and cardiovascular disease events or mortality	No	27/11	No	N/A	Yes/No	Statistical power	Yes
Kohler 2018^52^	Umbrella review of meta-analysis and MR studies	Environmental risk factors for depression	No	8/70	No	N/A	Yes/No	Proportion of variance in risk factors explained by genetic instruments	No
Kuźma 2018^a^,^24^^,^^53^	Systematic review	MR studies of risk factors and global cognitive function or dementia	Yes	18/0	Yes, Yes	Modified Q-Genie^25^	No	N/A	Yes
Li 2017^55^	Umbrella review of systematic reviews and meta-analyses	Serum uric acid level and multiple health outcomes	No	36/101	No	N/A	Yes/No	Statistical significance of the effect estimate and statistical power	Yes
Lor 2019^a^^,34^	Systematic review	MR analyses in oncological studies	Yes	77/0	No	N/A	No	N/A	Yes
Mamluk 2020^a^,^28^^,^^56^	Systematic review	Maternal alcohol consumption in pregnancy and offspring outcomes at birth and later in life	No	9/14	Yes, Yes	Self-developed tool	No	N/A	Yes
Markozannes 2021^57^	Umbrella review	C-reactive protein and health outcomes	No	37/55	Yes, No	Assessment of horizontal pleiotropy^b^	Yes/Yes	Statistical significance of the effect estimate	Yes
Meng 2019^58^	Systematic review of MR studies and MR analysis	MR studies of vitamin D and health outcomes	Yes	65/0	No	N/A	No	N/A	No
Pearson-Stuttard 2021^59^	Umbrella review	T2D and cancer incidence or mortality	No	8/20	Yes, No	Assessment of selection of genetic instrument	Yes/Yes	Statistical significance of the effect estimate	Yes
Pingault 2016^60^	Systematic review	MR studies of psychopathology-related outcomes	Yes	19/0	No	N/A	No	N/A	Yes
Riaz 2018^61^^,^^62^	Systematic review and meta-analysis of MR studies	MR studies of obesity and CVD	Yes	7/0	Yes, No	Evaluation of the three MR core assumptions	No	N/A	Yes
Robinson 2016^63^	Literature review on MR methods, applications and limitations and systematic review of MR studies	MR studies of rheumatology	Yes	33/0	No	N/A	No	N/A	Yes
Sommer 2018^64^	Systematic review	Childhood and adolescent obesity and future cardiovascular morbidity and mortality later in life	No	1/85	No	N/A	No	N/A	No
Swerdlow 2016^a^^,36^	Review on methods for selecting instruments for MR analysis and systematic review of MR studies	MR studies	Yes	231/0	No	N/A	No	N/A	Yes
Treur 2021^a^,^31^	Systematic review	MR studies of poor mental health and substance use	Yes	63/0	Yes, Yes	Self-developed tool	No	N/A	Yes
Vasta 2021^65^	Systematic review	Diabetes mellitus and amyotrophic lateral sclerosis	No	1/35	No	N/A	No	N/A	No
Yuan 2020^66^	Systematic review and MR analysis	MR studies of risk factors of T2D	Yes	40/0	No	N/A	No	N/A	No
Zhang X 2019^67^	Umbrella review	Non-genetic biomarkers and colorectal cancer	No	18/78	Yes, No	Assessment of horizontal pleiotropy	Yes/Yes	Statistical significance of the effect estimate, statistical power and evidence of bias due to directional pleiotropy	Yes
Zhang Z 2020^68^	Systematic review	Vitamin D and non-alcoholic fatty liver disease	No	1/12	No	N/A	No	N/A	No

a Included in the synthesis of tools for the assessing/evaluating MR studies.

b Based on the location of the SNPs. DNAm, DNA methylation; MR, Mendelian randomization; N/A, not applicable; SNP, single-nucleotide polymorphism; T2D, type 2 diabetes.

Of the 35 included reviews, 25 were systematic reviews and 10 were umbrella reviews. Of the 35 included reviews, 29 addressed a clinical question (i.e. included studies on the casual effect of an exposure vs an outcome) and 6 reviews addressed a methodological question (e.g. the status of reporting in MR studies); 17 reviews reported MR studies only and the other 18 reported both MR and non-MR studies; the number of MR studies ranged between 1 and 231 with a median of 18 studies. Of the 35 included reviews, 14 conducted an assessment of either risk of bias or quality of the evidence: 6 reviews conducted risk-of-bias assessments only, 5 reviews conducted quality-of-evidence assessments only and 3 did both. Details of the risk-of-bias and quality-of-evidence assessment in individual MR studies used in these 14 reviews are reported in Supplementary Table S4 (available as Supplementary data at *IJE* online).

A structured risk-of-bias tool for was used in five reviews: four of these (Grau-Perez,^22^ Kuźma,^24^ Mamluk^28^ and Treur^31^) used tools developed specifically for risk-of-bias assessment in MR studies that are included in the above sub-review of tools (see Supplementary Table S1, available as Supplementary data at *IJE* online, and Table 2); the fifth, Cheng,^45^ used the Newcastle-Ottawa Scale (NOS) for cohort studies,^69^ which was not specifically developed for MR studies. Four further reviews conducted risk-of-bias assessments but did not use a structured tool: Markozannes^57^ and X Zhang^67^ assessed horizontal pleiotropy; Pearson-Stuttard^59^ addressed the selection of the genetic instrument(s); and Riaz^61^^,^^62^ conducted evaluation of the three core assumptions.

Of the eight reviews that conducted a quality-of-evidence assessment, Markozannes^57^ and Pearson-Stuttard^59^ used a structured method based on statistical significance of the effect estimate and X Zhang^67^ used a structured method based on a combination of statistical significance of the effect estimate, statistical power and evidence of bias due to directional pleiotropy. Among the other five reviews in which a structured method was not used, Bochud^43^ based the assessment of quality of evidence on the strength of the genetic variant; Firth^47^ based the assessment on the results of the statistical analysis, the use of sensitivity analysis and test for bidirectional effects; Kim^51^ based the assessment on statistical power; Kohler^52^ based the assessment on the proportion of variance in risk factors explained by genetic instruments used; and Li^55^ based the assessment on the statistical significance of the effect estimate and the statistical power.

Of the 35 reviews included, 28 reported a general narrative description of potential bias and limitation in MR studies. Details of specific biases addressed narratively within these systematic reviews are reported in Supplementary Table S3 (available as Supplementary data at *IJE* online). Of these 28 reviews, 20 addressed bias related to the IV1 assumption (i.e. weak instrument bias), 16 reviews addressed bias related to the IV2 assumption (i.e. confounding, population stratification, assortative mating, dynastic effect and parent of origin effect)^14^ and 24 reviews addressed bias related to the IV3 assumption (i.e. horizontal pleiotropy). In addition, 17 reviews addressed bias related to the selection of the genetic instrument (i.e. linkage disequilibrium, Winner’s course bias, segregation distortion, monotonicity and homogeneity), 6 reviews addressed bias related to the selection of the population or sample (i.e. population heterogeneity and selection bias), 8 reviews addressed bias due to canalization and 4 reviews addressed bias due to measurement errors or misclassification. In addition to bias, we also evaluated whether other MR-relevant topics were narratively described and we found that 11 reviews addressed precision of the results (i.e. low statistical power or sample size), 5 reviews addressed reverse causation (or bidirectionality), 3 reviews addressed the inability to assess non-linear associations, 2 reviews addressed statistical analysis and lack of genetic instrument, respectively, and 1 review addressed inability to assess dose–response estimations.

#### Protocols for systematic reviews

**Figure 3:**
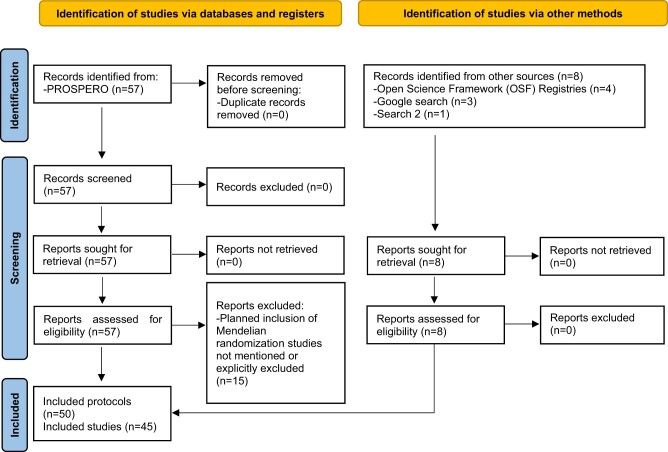
Flow diagram of identification, screening and inclusion of protocols of systematic reviews (and meta-analysis) planning to include Mendelian randomization studies

Our final search for protocols of systematic reviews (Search 3) identified 65 protocols (57 from database searches and 8 from other searches, including 1 from Search 2) of which 15 were excluded because inclusion of MR studies was not specified or MR studies were specified in the exclusion criteria. A flow diagram of identification, screening and inclusion of protocols of systematic reviews is shown in Figure 3. Two protocols for the same review were identified from different sources for five reviews so a total of 45 study protocols were included in this part of the review. A list of included protocols with details of the method used by each of study is reported in Table 4 and the 15 protocols that did not meet the criteria for inclusion are listed in Supplementary data (available as Supplementary data at *IJE* online). Five of the 45 included protocols were of published systematic reviews that were included in our sub-review of systematic reviews above.^90^^,^^97^^,^^110^^,^^116^^,^^117^ Of the 45 included protocols, 35 were for systematic reviews of primary studies and 10 were for umbrella reviews. Fifteen protocols were for reviews of MR studies only and 30 planned to include other study designs.

**Table 4:** List of included protocols of systematic reviews reporting Mendelian randomization studies Newcastle-Ottawa Scale^21^

Study ID	Topic of the review	Type of study	MR studies only?	Is there a plan to assess for risk of bias/quality of evidence in MR studies? If Yes, is a structured tool/approach used?	What approach/method/tool?
Ansu 2020^70^	Whole blood ionized magnesium in healthy adults	Systematic review	No	No	N/A
Baldwin 2020^71^	The impact of childhood maltreatment on mental health	Systematic review and meta-analysis	No	NS/Yes	Adapted version of the Newcastle-Ottawa Scale^69^
Cara 2020^72^	Safety of enteral nutrition formulations with dietary fibre	Systematic review	No	NS/Yes	Adapted version of the Newcastle-Ottawa Scale^69^
Cheng 2019b,^117^	Puberty timing and T2D	Systematic review	No	NS/Yes	Newcastle-Ottawa Scale^69^
Dack 2020^73^	Early-life exposure to mercury, growth and neurodevelopment	Systematic review	No	NS/Yes	Newcastle-Ottawa Scale^69^
Desai 2021^74^	Risk factors for dementia	Systematic review	Yes	Yes/Yes	Q-Genie^25^
Elsakloul 2016^75^	Serum uric acid and cardiovascular diseases	Systematic review	No	NS/Yes	Pre-specified bespoke tool based on STROBE^118^
Fan 2020^76^	Habitual coffee consumption and lung function decline	Systematic review	No	NS/Yes	Tool for systematic reviews of observational studies that comprised four key domains: external validity, reporting, bias and confounding factors (no reference provided)
Fell 2020^77^	Maternal smoking and orofacial clefts	Systematic review and meta-analysis	No	NS/Yes	Newcastle-Ottawa Scale^69^
Gianfredi 2019^78^	Physical activity and depression	Systematic review	No	NS/Yes	Adapted version of the Newcastle-Ottawa Scale^69^
Gibson 2021^79^	Reporting quality in MR studies using UK Biobank data	Systematic review of MR studies	Yes	No	N/A
Grover 2018^80^^,^^81^	Risk factors for neurodegenerative diseases	Systematic review of MR studies	Yes	Yes/No	Assessment and reporting of MR studies based on previous published method protocol by Grover at al.^27^
Haan 2018^82^^,^^83^	Alcohol, tobacco and caffeine consumption in pregnancy and externalizing disorders in offspring	Systematic review	No	NS/Yes	Newcastle-Ottawa Scale^69^
Ibrahim 2020^84^	MR studies of abdominal aortic aneurysms	Systematic review of MR studies and meta-analysis	Yes	Yes/Yes	STROBE-MR^23^^,^^85^ and other publications
Jiang 2019^86^	Causal factors associated with risk or survival in lung cancer	Systematic review of MR studies	Yes	Yes/No	Assessment of risk of bias and quality of reporting of MR studies based on previous method protocol by Grover *et al.*^27^. Assessment of the robustness and credibility of the data synthesis using sensitivity analysis
Julian 2020^87^	MR studies of neurodegenerative disease	Systematic review of MR studies	Yes	Yes/No	Assessment of risk of bias, evidence base for methodological strengths and weaknesses using the published literature (no reference provided)
Karwatowska 2020^88^^,^^89^	Risk factors for disruptive behaviours	Systematic review and meta-analysis	No	NS/Yes	Adapted version of the ROBINS-I checklist^19^
Kim 2020a^90^	Obesity and cardiovascular outcomes	Umbrella review	No	No	N/A
Kim 2020b^91^	Obesity and gastroenterological diseases	Umbrella review	No	No	N/A
Kim 2021^b^^,92^	Obesity and renal and genitourinary outcomes	Umbrella review	No	No	N/A
Lee LS 2020^a^^,26^	Risk factors for dementia	Systematic review of MR studies and meta-analysis	Yes	Yes/No	Assessment of quality using a self-developed questionnaire based on published guidelines
Lee M 2018^93^	MR studies using adiposity as an exposure	Systematic review of MR studies	Yes	Yes/Yes	Descriptive assessment of choice of methods and genetic variants used in included studies
Lemus 2021^94^	T2D and incidence of 17 types of cancer	Systematic review and meta-analysis	No	NS/Yes	Newcastle-Ottawa Scale^69^
Liu 2020^95^	Risk factors for coronavirus disease 19 (COVID-19)	Umbrella review	No	No	N/A
Luo 2017^96^	MR studies compared with randomized–controlled trials	Systematic review	No	Yes/No	Assessment of the robustness and credibility of an estimate based on sensitivity analysis methods and different choices of genetic variants as instrumental variables
Mamluk 2015^b^^,97^	Prenatal alcohol exposure on pregnancy and childhood outcomes	Systematic review	No	NS/No	Assessment of quality of evidence based on whether studies have adjusted for smoking and maternal education/social class as potential confounders in their final model
Maretzke 2018^c^^,98^	Role of vitamin D in preventing and treating selected extra-skeletal diseases	Umbrella review	No	No	N/A
Markozannes 2021^99^	Genetically predicted risk factors associated with cancer risk	Systematic review of MR studies	Yes	Yes/Yes	Self-developed tool based on the results of the main analysis and of the sensitivity analysis
Naassila 2021^100^	Alcohol intake and risk of cardiovascular diseases	Systematic review	No	Yes/Yes	Q-Genie^25^
Naassila 2021^101^	Alcohol intake and risk of neurological diseases	Systematic review	No	Yes/Yes	Q-Genie^25^
Naassila 2021^102^	Alcohol intake and cancers, neurological, cardiovascular and liver diseases	Systematic review	Yes	Yes/Yes	Q-Genie^25^
Romo 2018^103^	Conduct and reporting of MR studies	Systematic review of MR studies	Yes	No	N/A
Saribaz 2020^104^	Environmental risk factors of child and adolescents’ depressive and anxious psychopathology	Systematic review	No	Yes/NR	Self-developed method developed at the time of review
Shi 2020^105^^,^^106^	Prenatal alcohol exposure and offspring health outcomes	Umbrella review	No	Yes/Yes	Modified recently developed tool (reference not provided)
Solmi 2018a^107^	Safety and efficacy of cannabinoids and cannabis in treating medical conditions	Umbrella review	No	No	N/A
Solmi 2018b^108^	Psychosis and non-communicable general medical conditions	Umbrella review	No	No	N/A
Suh 2021^109^	Risk factors for cardiovascular multimorbidity	Systematic review	No	NS/Yes	Newcastle-Ottawa Scale^69^
Treur 2019^b^^,110^	Substance use, cognitive functioning and psychiatric disorders	Systematic review of MR studies	Yes	Yes/No	Descriptive assessment based on MR study design, choice of genetic variants, whether there was sample overlap in the case of two-sample MR studies and the use of sensitivity analyses
van Oort 2020^111^	Alcohol consumption and its causal relationship with mortality, cardiometabolic diseases and risk factors	Systematic review of MR studies	Yes	Yes/No	Assessment of the quality of MR studies based on previous published method protocol by Grover *et al.*^27^ with focus on MR design, the quality of the genetic instrument and the validation of the MR assumptions
Verdiesen 2021^85^	Causal risk factors for breast cancer	Systematic review of MR studies and meta-analysis	Yes	Yes/Yes	STROBE-MR and a published checklist by Davies *et al.*^21^
Visontay 2020^112^^,^^113^	Alcohol consumption and health outcomes	Systematic review	No	Yes/Yes	Recently developed risk-of-bias tools specific to MR studies, natural experiments, and other genetic-based methods by Mamluk *et al.*^28^
Wang 2018^114^	Vitamin D deficiency as a causal risk factor	Umbrella review	No	NS/Yes	Assessment of risk of bias as described in the Cochrane risk-of-bias tool (reference not provided)
Wong 2021^115^	Factors contributing to higher coronavirus disease 19 (COVID-19) risk or its severity	Living systematic review	Yes	Yes/Yes	Assessment of risk of bias based on a published checklist by Davies *et al.*^21^
Yan 2020^54^	Metabolomic profiling of amino acids in serum/plasma and urine and risk of cardiovascular disease and T2D	Systematic review and meta-analysis	No	NS/Yes	Assessment of risk of bias using the Cochrane risk-of-bias tool (for randomized–controlled trials)^18^ and the ROBINS-I^19^
Zhang 2018^b^^,116^	Non-genetic biomarkers and risk of colorectal cancer	Umbrella review	No	No	N/A

a Included in the synthesis of tools for assessing, conducting and reporting MR studies.

b Protocols of published systematic reviews included in this article.

c Protocol of published systematic review not included in this article. MR, Mendelian randomization; N/A, not applicable; NR, not reported; NS, non-specifically; ROBINS-I, risk of bias in non-randomized studies of intervention; STROBE, Strengthening the Reporting of Observational Studies in Epidemiology; T2D, type 2 diabetes.

Eighteen protocols reported plans for a MR-specific risk-of-bias/quality-of-evidence assessment and 15 protocols reported plans for a non-MR-specific risk-of-bias/quality-of-evidence assessment. Of the 18 protocols with a MR-specific risk-of-bias/quality-of-evidence assessment, the use of a structured tool/method was planned in 11 protocols, the use of other methods/approaches was planned in 12 protocols and 1 protocol described the use of a method that the author planned to develop at the time of conducting the review. Of the 11 protocols describing use of a structured tool, Ibrahim^84^ and Verdiesen^85^ planned to use STROBE-MR^32^^,^^33^ and other published literature, including the MR guidelines by Davies,^21^ LS Lee^26^ planned to use a self-developed questionnaire (also included in our synthesis of tools) based on published guidelines including Davies,^21^ Grover^27^ and Burgess.^20^ Markozannes^99^ planned to use a self-developed tool based on the results of the main analysis and of the sensitivity analysis; Naassila^100–102^ planned to use Q-GENIE;^25^ Shi^105^^,^^106^ planned to use a modified version of a recently developed tool (no reference provided); Visontay^112^^,^^113^ planned to use the tool developed by Mamluk;^28^ and Wong^115^ planned to conduct risk-of-bias assessment based on the guidelines from Davies.^21^ Of the seven protocols describing a MR-specific risk-of-bias/quality-of-evidence assessment without using a structured tool, four planned an assessment based on the literature: Grover,^80^^,^^81^ Jiang^86^ and van Oort^111^ referred to the MR methods protocol published by Grover^27^ and Julian^87^ did not report any reference. Of the remaining four protocols, Saribaz^104^ planned to develop a risk-of-bias assessment method at the time of conducting the review; M Lee^93^ planned to perform a descriptive assessment of the MR methods and of the genetic variants used in included studies; Luo^96^ planned to perform an assessment based on sensitivity analysis methods and different choices of genetic variants as instrumental variables; Treur^110^ planned to perform an assessment based on sensitivity analysis methods, on the choice of genetic variants, on the presence of sample overlap (two-sample MR studies) and on the use of sensitivity analyses.

Of the 15 protocols in which a non-MR-specific risk-of-bias assessment is reported, 14 used structural tools and Mamluk^97^ planned to assess risk of bias on whether adjustment for potentially relevant confounders was conducted. Of the 14 structured tools used for non-MR-specific risk-of-bias assessment, Cheng,^117^ Dack,^73^ Fell,^77^ Haan,^82^^,^^83^ Lemus^94^ and Suh^109^ planned to use NOS^69^ and Baldwin,^71^ Cara^72^^,^^109^ and Gianfredi^78^ planned to use a modified version of NOS; Elsakloul^75^ planned to use STROBE;^118^ Fan^76^ planned to use a quality-assessment tool for systematic reviews of observational studies that comprised external validity, reporting, bias and confounding factors, but a reference was not provided; Karwatowska^88^^,^^89^ planned to use ROBINS-I;^19^ Yan^54^ planned to use the ROB-2^18^ and the ROBINS-I^19^ tools; and Wang^114^ planned to use the Cochrane risk-of-bias assessment tool (no details provided).

## Discussion

Our systematic review of tools identified 14 instruments developed for the evaluation, conduct and/or reporting of MR studies. Half of the tools were designed (or used) either entirely or partially for the evaluation of MR studies. Most of these tools were developed for application within a systematic review,^22^^,^^24^^,^^26^^,^^28^^,^^31^ whereas only two were developed for general use.^20^^,^^21^ Despite notable variability in the structure and content of the evaluating tools, all tools contained items addressing the validity of the three core IV assumptions. In addition, all but one of the tools addressed bias related to the selection of the population(s) or sample(s), including population heterogeneity, sample overlap, choice of controls and selection bias. Just over half of the tools addressed bias related to the genetic instrument, including linkage disequilibrium, construct of the genetic score and lack of variants harmonization, and addressed the conduct of sensitivity analysis. Fewer than half of the evaluating tools addressed bias due to measurement errors and only one tool addressed bias due to other sources including missing data. Although it was not in our scope to critically appraise the identified tools, by compiling a list and inspecting the content of these tools we found that all tools, including these designed for reporting and conducting, addressed these assumptions or conditions within the MR analysis that, when violated, lead to potential bias of the MR causal estimate. Of the seven tools designed (or used) for evaluation of MR studies, three tools included a scoring/rating system^24^^,^^28^^,^^31^ but none of the tools attempted to predict the likely direction of bias (i.e. whether the results are biased away from or towards the null).

Consistently with the lack of formal tools for assessment of risk of bias in MR studies, only a small proportion (26%) of the systematic reviews of MR studies included in our review conducted a risk-of-bias assessment and only 23% of the included reviews conducted an assessment of evidence of causal effect within individual MR studies. Nevertheless, most of the reviews included a narrative description of MR-related bias and limitations (74%) and—as observed in the content of the tools—among these, most of the reviews addressed bias related to the core IV assumptions of relevance (IV1) and exclusion restriction (IV3) (71% and 86%, respectively), but only 57% addressed bias related to the independence assumption (IV2), whereas 61% addressed bias related to the genetic instrument and only 21% addressed bias related to the selection of the population or sample.

In contrast with published systematic reviews, when we looked at protocols of systematic reviews of (or including) MR studies, a plan to conduct an assessment was reported in 73% of the protocols included in our reviews, although only in 40% was the approach or methodology used specific for MR studies. This higher proportion may reflect an increased focus on risk of bias over time or may reflect a tendency for review teams who publish their protocols to include risk-of-bias assessments in their plans. Of protocols that specified methodologies specific to MR studies, only 39% planned to use a structured tool, including the STROBE-MR,^32^^,^^33^ Q-GENIE,^25^ a self-developed tool included in our synthesis of tools^26^ and a tool developed within another systematic review.^28^ One review protocol planned to use a recently developed tool that, similarly to the tool developed by Mamluk,^28^ consisted of five questions, one for bias domain, including instrument bias, genetic confounding and selection bias. The rest of the protocols not planning to use a structured tool proposed other informal ways to address bias, including assessment based on the validation of the three IV core assumptions, the choice of genetic instruments, the use of sensitivity analysis and description of MR analysis design, and some of these approaches were based on MR literature including MR guidelines by Davies^21^ and Grover.^27^

Our review has strengths and limitations. First, we included published and unpublished articles by searching several relevant databases for peer-reviewed articles, preprints archives and Google Scholar for preprints articles and unpublished studies. Furthermore, we developed specific search strings for each objective with the assistance of an information specialist. However, as some of the tools we have identified were developed within other types of articles, including literature reviews and systematic reviews of MR and non-MR studies, it is possible that our searches may have missed some tools. As data extraction was performed by a single author, it is possible that some errors in data collection were made. Our classification of items into bias domains and specific issues is to an extent arbitrary and some items could have been classified in accordance with more than one bias or limitation. For example, we classified linkage disequilibrium as relevant to the choice of genetic variant because it mainly introduces horizontal pleiotropy^17^ (IV3 domain) although it has been argued to be associated also with confounding (IV2 domain).^5^

By summarizing the currently available knowledge on methods and approaches for assessment of risk of bias in MR studies, our longer-term aim was to identify potential items for inclusion in a structured tool for risk-of-bias assessment in MR studies. We are not able to make a recommendation on what tool(s) should be adopted to assess MR studies, as none of the tools identified by our searches appears to have been formally tested or validated. A systematic process to test reliability using formal studies of agreement should be conducted before the tools can be recommended for general use. Validation studies could in theory be undertaken using a meta-epidemiological approach, in which effect estimates are compared between studies with different bias-related features. However, such studies require large numbers of meta-analyses with their included studies all having been assessed using the same tool(s).

Nevertheless, the content of the tools that we have identified in our review will be a useful source of information on what bias/limitations reviewers should be aware of when conducting a systematic review (and meta-analysis) including results from MR studies. This suggests that issues to address include those arising from departures from the IV assumptions, those related to the choice of genetic instrument(s) and those arising from the population from which the data are collected (particularly in two-sample MR), in addition to more traditional non-MR-specific epidemiological biases.

## Ethics approval

Not applicable. All the work was developed using published data.

## Supplementary Material

dyac149_Supplementary_Data

## Data Availability

All materials used in this study are available in the Supplementary material, available as Supplementary data at *IJE* online, or the main text.
