# Supplementary material for: Tools for assessing quality and risk of bias in Mendelian randomization studies: a systematic review
Source: Int J Epidemiol. 2022 Jul 28;52(1):227–49. doi: 10.1093/ije/dyac149 (PMC9908059; doi:10.1093/ije/dyac149)
Supplement: dyac149_Supplementary_Data [file dyac149_supplementary_data.docx]

**Supplementary data**

1. Search strategy for tools aimed at evaluation of the design, conduct and/or reporting of Mendelian randomization studies (search 1)
2. Search strategy for systematic reviews of Mendelian randomization studies (search 2)
3. Search strategy for protocols of systematic reviews of Mendelian randomization studies (search 3)
4. Studies excluded at full text with exclusion reasons
5. Supplementary Table 1: List of included studies containing one or more tools for evaluating, conducting, and reporting MR studies.
6. Supplementary Table 2: Details of other MR-relevant content of items or questions within each assessing tool.
7. Supplementary Table 3: Details of reporting and conducting tools
8. Supplementary Table 4: Details of bias addressed by a narrative description in systematic reviews of MR studies.

**1. Search strategy for tools aimed at evaluation of the design, conduct and/or reporting of Mendelian randomization studies (search 1)**

**Ovid MEDLINE ALL and Embase**

1. Mendelian randomi#ation.hw,tw,kw,kf.

2. genetic instrument?.hw,tw,kw,kf.

3. (genetic variant* adj3 instrumental variable*).tw,kw,kf.

4. or/1-3

5. scale*. tw,kw,kf.

6. checklist*. tw,kw,kf.

7. critical apprais*.tw,kw,kf.

8. tool*. tw,kw,kf.

9. guide*. tw,kw,kf.

10. (Dictionary or glossary). tw,kw,kf.

11. or/5-10

12. valid*. hw,tw,kw,kf.

13. quality. hw,tw,kw,kf.

14. ((bias OR confound*) AND (asses* OR measur* OR evaluat*)).tw,kw,kf.

15. or/12-14

16. 4 and 11 and 15

Fields: *hw* ([subject] heading word); *kf* (keyword heading word (MEDLINE)); *kw* (keyword (Embase); keyword heading (MEDLINE)); tw (text word).

**Web of Science**

(**TI**=((("Mendelian randomization" or "Mendelian randomisation" or "genetic instrument*" or ("genetic variant*" and "instrumental variable*")) AND (scale* or checklist* or “critical apprais*” or tool* or guide* or dictionary or glossary) AND (valid* or quality or ((bias or confound*) and (asses* or measur* or evaluat*)))))) OR

(**AB**=((("Mendelian randomization" or "Mendelian randomisation" or "genetic instrument*" or ("genetic variant*" and "instrumental variable*")) AND (scale* or checklist* or “critical apprais*” or tool* or guide* or dictionary or glossary) AND (valid* or quality or ((bias or confound*) and (asses* or measur* or evaluat*)))))) OR (**AK**=((("Mendelian randomization" or "Mendelian randomisation" or "genetic instrument*" or ("genetic variant*" and "instrumental variable*")) AND (scale* or checklist* or “critical apprais*” or tool* or guide* or dictionary or glossary) AND (valid* or quality or ((bias or confound*) and (asses* or measur* or evaluat*))))))

Fields: **TI**: title; **AB**: abstract; **AK**: author keyword;

**2. Search strategy for systematic reviews of Mendelian randomization studies (search 2)**

**Ovid MEDLINE ALL and Embase**

1. Mendelian randomi#ation.hw,tw,kw,kf.

2. genetic instrument?.hw,tw,kw,kf.

3. (genetic variant* adj3 instrumental variable*).tw,kw,kf.

4. or/1-3

5. ((systematic or meta) adj2 (analys* or review)).ti,kw,kf.

6. ((systematic* or quantitativ* or methodologic*) adj5 (review* or overview*)).ti,ab,kw,kf,sh.

7. (quantitativ$ adj5 synthesis$).ti,ab,kw,kf,hw.

8. meta-analysis.pt.

9. (meta-analys* or meta analys* or metaanalys* or meta synth* or meta-synth* or metasynth*).ti,ab,kw,kf,hw.

10. ((umbrella or overview) adj3 (review? or meta-analys*)).mp.

"review of meta-analyses".mp.

11. (2046-4053).is. [ISSN for Systematic Reviews journal]

12. or/ 5-13

13. 4 and 14

Fields: *hw* ([subject] heading word); *kf* (keyword heading word (MEDLINE)); *kw* (keyword (Embase); keyword heading (MEDLINE)); *tw* (text word); *ti* (title); *ab* (abstract); *mp* (multiple places search – which also includes *ot* (original title); *ox* (other index terms word (Embase))); *is* (ISSN prints (MEDLINE); ISSN (Embase)).

**Web of Science**

(**TI**=(("Mendelian randomization" or "Mendelian randomisation" or "genetic instrument*" or ("genetic variant*" and "instrumental variable*")) AND (("systematic or meta" NEAR "analys* or review") or (systematic* or quantitativ* or methodologic* NEAR review* or overview*) or (meta-analys* or meta analys* or metaanalys* or meta synth* or meta-synth* or metasynth*)))) OR

(**AB**=(("Mendelian randomization" or "Mendelian randomisation" or "genetic instrument*" or ("genetic variant*" and "instrumental variable*") ) AND (("systematic or meta" NEAR "analys* or review") or (systematic* or quantitativ* or methodologic* NEAR review* or overview*) or (meta-analys* or meta analys* or metaanalys* or meta synth* or meta-synth* or metasynth*)))) OR

(**AK**=(("Mendelian randomization" or "Mendelian randomisation" or "genetic instrument*" or ("genetic variant*" and "instrumental variable*") ) AND (("systematic or meta" NEAR "analys* or review") or (systematic* or quantitativ* or methodologic* NEAR review* or overview*) or (meta-analys* or meta analys* or metaanalys* or meta synth* or meta-synth* or metasynth*))))

Fields: **TI**: title; **AB**: abstract; **AK**: author keyword;

**bioRxiv** and **medRxiv**: Mendelian AND randomi*ation AND review

**3. Search strategy for protocols of systematic reviews of Mendelian randomization studies (search 3):**

**PROSPERO**: Mendelian randomization OR Mendelian randomisation

**4. Studies excluded at full text with exclusion reasons**

**Tools aimed at evaluation of the design, conduct and/or reporting of Mendelian randomization studies (search 1)**

Conference abstract not linked to an included full text article (1 reference):

1. Frater J, McGrath J. Mendelian randomization in psychiatry: A guide for clinicians in understanding the latest in epidemiological genetic research. Aust N Z J Psychiatry. 2019; 53 (Supplement 1): 33.

Not a tool (5 references):

1. Gala H, Tomlinson I. The use of Mendelian randomisation to identify causal cancer risk factors: promise and limitations. J Pathol. 2020; 250(5): 541-54.
2. Grover S, Del Greco F, Konig IR. Evaluating the current state of Mendelian randomization studies: A protocol for a systematic review on methodological and clinical aspects using neurodegenerative disorders as outcome. Syst Rev. 2018; 7(1): 145.
3. Haycock PC, Burgess S, Wade KH, Bowden J, Relton C, Smith GD. Best (but oft-forgotten) practices: the design, analysis, and interpretation of Mendelian randomization studies. Am J Clin Nutr 2016; 103(4): 965-78.
4. Pagoni P, Dimou NL, Murphy N, Stergiakouli E. Using Mendelian randomisation to assess causality in observational studies. Evid Based Ment Health 2019; 22(2): 67-71.
5. Teumer A. Common Methods for Performing Mendelian Randomization. Front Cardiovasc Med 2018; 5: 51.

**Systematic reviews of Mendelian randomization studies (search 2)**

Conference abstract not linked to an included full text article (14 references):

1. Aragam KG, Zhao W, Shah O, et al. Serum Gamma glutamyl transferase and risk of coronary artery disease: A causal assessment by mendelian randomization. Circulation 2013; 128(22).
2. Banach M, Katsiki N, Mazidi M. SLEEP DURATION MAY NOT HAVE ANY EFFECT ON THE RISK OF STROKE: INSIGHTS FROM MENDELIAN RANDOMIZATION AND PROSPECTIVE COHORT STUDIES. J Am Coll Cardiol 2020; 75(11): 2038-None.
3. 3Choi J, McCormick N, Choi H, et al. The impact of genetically determined serum urate levels on the development of cardiovascular diseases: A systematic review and meta-analysis of mendelian randomization studies. Ann Rheum Dis 2020; 79: 865-6.
4. Forouhi NG, Ye Z, Rickard AP, et al. The association and causal inference between circulating 25-hydroxy vitamin D concentration and the risk of type 2 diabetes. Diabetologia 2011; 54: S75.
5. Haring R. Does testosterone shorten life? Triangulation of a much-debated association. BMJ Evid Based Med 2018; 23: A26.
6. Henderson J. The impact of obesity and infant growth patterns on childhood wheezing. Pediatr Pulmonol 2017; 52: S88-S9.
7. Kamel S, Richards B, Dufresne L, Engert J, Thanassoulis G. Total cholesterol, low-density lipoprotein cholesterol, or high density lipoprotein and bone health: Systematic review and Mendelian randomization. Can J Cardiol 2015; 31(10): S304.
8. Lee MA, Wade KH, Corbin LJ, Timpson NJ. Do causal estimates of differential adiposity effects show evidence of impact on the circulating metabolome? Genet Epidemiol 2020; 44(5): 498.
9. Marjot T, Yadav S, Sharma P. The genetics of cerebral sinovenous thrombosis; a meta-analysis of > 2000 CSVT patients and > 9000 controls. Int J Stroke 2010; 5: 45.
10. Mazidi M, Dehghan A, Mikhailidis DP, et al. The Association between Coffee and Caffeine Consumption and Renal Function: Insight from Individual-Level Data, Mendelian Randomization, and Meta-Analysis. Arch Med Sci 2022;18(4)
11. Morris H. Vitamin D for conditions other than bone disease: An update of the evidence. Clinica Chimica Acta 2019; 493: S733.
12. Tsangaris I, Tsaknis G, Nikolopoulos G, et al. The association between circulating plasminogen activator inhibitor type 1 (PAI-1) levels, the PAI-1 4g/5g polymorphism and incident myocardial infarction. Intensive Care Med 2013; 39: S245-S6.
13. Wang N, Rodgers A. 471 Cumulative Benefits of Cholesterol Lowering for the Prevention of Cardiovascular Events. Heart Lung Circ 2020; 29: S249.
14. Zittermann A. Vitamin D and cardiovascular disease. Anticancer Res 2019; 39(6): 3289-90.

Not a systematic review (43 references):

1. Benn M, Nordestgaard Bo G. From genome-wide association studies to Mendelian randomization: Novel opportunities for understanding cardiovascular disease causality, pathogenesis, prevention, and treatment. Cardiovasc Res 2018; 114(9): 1192-208.
2. Berry D, Hypponen E. Determinants of vitamin D status: focus on genetic variations. Curr Opin Nephrol Hypertens 2011; 20(4): 331-6.
3. Bloomgarden Z. Diabetes and branched-chain amino acids: What is the link? J Diabetes 2018; 10(5): 350-2.
4. Brunner C, Davies NM, Martin RM, et al. Alcohol consumption and prostate cancer incidence and progression: A Mendelian randomisation study. Int J Cancer 2017; 140(1): 75-85.
5. Francischetti EA, Dezonne RS, Pereira CM, et al. Insights into the controversial aspects of adiponectin in cardiometabolic disorders. Horm Metab Res 2020; 52(10): 695-707.
6. Franks PW, Atabaki-Pasdar N. Causal inference in obesity research. J Intern Med 2017; 281(3): 222-32.
7. Franks PW, Timpson NJ. Genotype-Based Recall Studies in Complex Cardiometabolic Traits. Circ Genom Precis Med 2018; 11(8): e001947.
8. Grover S, Del Greco F, Konig IR. Evaluating the current state of Mendelian randomization studies: A protocol for a systematic review on methodological and clinical aspects using neurodegenerative disorders as outcome. Syst Revs 2018; 7(1): 145.
9. Hiemstra TF, Lim K, Thadhani R, Manson JE. Vitamin D and Atherosclerotic Cardiovascular Disease. J Clin Endocrinol Metab 2019; 104(9): 4033-50.
10. Jansen H, Lieb W, Schunkert H. Mendelian Randomization for the Identification of Causal Pathways in Atherosclerotic Vascular Disease. Cardiovasc Drugs Ther 2016; 30(1): 41-9.
11. Jansen H, Samani NJ, Schunkert H. Mendelian randomization studies in coronary artery disease. Eur Heart J 2014; 35(29): 1917-24.
12. Julian TH, Glascow N, Barry ADF, et al. Physical exercise is a risk factor for amyotrophic lateral sclerosis: Convergent evidence from Mendelian randomisation, transcriptomics and risk genotypes. EBioMedicine 2021; 68: 103397.
13. Kabat GC, Hosgood HD, Rohan TE. Adult Height in Relation to the Incidence of Cancer at Different Anatomic Sites: the Epidemiology of a Challenging Association. Curr Nutr Rep 2016; 5(1): 18-28.
14. Kelley GA, Kelley KS, Stauffer BL. Obesity and cardiovascular outcomes: another look at a meta-analysis of Mendelian randomization studies. J Investig Med 2020; 68(2): 357-63.
15. Larsson SC. Mendelian randomization as a tool for causal inference in human nutrition and metabolism. Curr Opin Lipidol 2021; 32(1): 1-8.
16. Lega IC, Lipscombe LL. Review: Diabetes, Obesity, and Cancer-Pathophysiology and Clinical Implications. Endocr Rev 2020; 41(1): bnz014.
17. Lim KX, Rijsdijk F, Hagenaars SP, et al. Studying individual risk factors for self-harm in the UK Biobank: A polygenic scoring and Mendelian randomisation study. PLoS Med 2020; 17(6): e1003137.
18. Maretzke F, Bechthold A, Egert S, et al. Role of Vitamin D in Preventing and Treating Selected Extraskeletal Diseases-An Umbrella Review. Nutrients 2020; 12(4).
19. Meyer HV, Dawes TJW, Serrani M, et al. Genetic and functional insights into the fractal structure of the heart. Nature 2020; 584(7822): 589-94.
20. Mokry LE, Ahmad O, Forgetta V, Thanassoulis G, Richards JB. Mendelian randomisation applied to drug development in cardiovascular disease: A review. J Med Genet 2015; 52(2): 71-9.
21. Nelson CP, Erridge C. Are toll-like receptors potential drug targets for atherosclerosis? Evidence from genetic studies to date. Immunogenetics 2019; 71(1): 1-11.
22. O'Mara TA, Glubb DM, Kho PF, Thompson DJ, Spurdle AB. Genome-wide association studies of endometrial cancer: Latest developments and future directions. Cancer Epidemiol Biomarkers Prev 2019; 28(7): 1095-102.
23. Okuyama H, Hamazaki T, Hama R, et al. A Critical Review of the Consensus Statement from the European Atherosclerosis Society Consensus Panel 2017. Pharmacology 2018; 101(3): 184-218.
24. Pierce BL, Kraft P, Zhang C. Mendelian randomization studies of cancer risk: a literature review. Curr Epidemiol Rep 2018; 5(2): 184-96.
25. Polimanti R, Peterson RE, Ong JS, et al. Evidence of causal effect of major depression on alcohol dependence: findings from the psychiatric genomics consortium. Psychol Med 2019; 49(7): 1218-26.
26. Sallis H, Davey Smith G, Munafo MR. Genetics of biologically based psychological differences. Philos Trans R Soc Lond B Biol Sci 2018; 373(1744).
27. Schooling CM. Testosterone and cardiovascular disease. Curr Opin Endocrinol Diabetes Obes 2014; 21(3): 202-8.
28. Shah NP, Pajidipati NJ, McGarrah RW, et al. Lipoprotein (a): An Update on a Marker of Residual Risk and Associated Clinical Manifestations. Am J Cardiol 2020; 126: 94-102.
29. Stewart DJ, Langlois V, Noone D. Hyperuricemia and hypertension: Links and risks. Integr Blood Press Control. 2019; 12: 43-62.
30. Storm CS, Kia DA, Almramhi M, Wood NW. Using Mendelian randomization to understand and develop treatments for neurodegenerative disease. Brain Commun 2020; 2(1): fcaa031.
31. Swerdlow DI, Hingorani AD, Humphries SE. Genetic Risk Factors and Mendelian Randomization in Cardiovascular Disease. Curr Cardiol Rep 2015; 17(5).
32. Swerdlow DI, Preiss D. Genetic insights into statin-associated diabetes risk. Curr Opin Lipidol 2016; 27(2): 125-30.
33. Tanner-Smith EE, Egger M, Higgins J. Systematic reviews in health research. Res Synth Methods 2019; 10(3): 310-1.
34. Verzilli C, Shah T, Casas JP, et al. Bayesian meta-analysis of genetic association studies with different sets of markers. Am J Hum Genet 2008; 82(4): 859-72.
35. Waubant E, Lucas R, Mowry E, et al. Environmental and genetic risk factors for MS: an integrated review. Ann Clin Transl Neurol 2019; 6(9): 1905-22.
36. Winning L, Linden GJ. Periodontitis and Systemic Disease: Association or Causality? Curr Oral Health Rep 2017; 4(1): 1-7.
37. Yarmolinsky J, Wade KH, Richmond RC, et al. Causal Inference in Cancer Epidemiology: What Is the Role of Mendelian Randomization? Cancer Epidemiol Biomarkers Prev 2018; 27(9): 995.
38. Yarmolinsky J, Wade KH, Richmond RC, et al. Causal inference in cancer epidemiology: what is the role of Mendelian randomization? bioRxiv 223966; doi: <https://doi.org/10.1101/223966>, preprint: not peer reviewed.
39. Zheng J, Zhang Y, Liu Y, et al. Multi-omics study revealing tissue-dependent putative mechanisms of SARS-CoV-2 drug targets on viral infections and complex diseases medRxiv 2020.05.07.20093286; doi: https://doi.org/10.1101/2020.05.07.20093286, preprint: not peer reviewed.
40. Zhou Y, Liu Z, Yang H, et al. Coagulation factors and COVID-19 severity: Mendelian randomization analyses and supporting evidence. medRxiv 2020.11.20.20235440; doi: <https://doi.org/10.1101/2020.11.20.20235440>, preprint: not peer reviewed.
41. Zhu Z, Hasegawa K, Camargo CA, Liang L. Investigating asthma heterogeneity through shared and distinct genetics: Insights from genome-wide cross-trait analysis. J Allergy Clin Immunol 2021; 147(3):796-807.
42. Zittermann A. Vitamin D status, supplementation and cardiovascular disease. Anticancer Res 2018; 38(2): 1179-86.
43. Zittermann A, Pilz S. Vitamin D and cardiovascular disease: An update. Anticancer Res 2019; 39(9): 4627-35.

Do not include Mendelian randomization studies (46 references):

1. Belbasis, L., M.C. Mavrogiannis, M. Emfietzoglou, and E. Evangelou. Environmental factors, serum biomarkers and risk of atrial fibrillation: an exposure-wide umbrella review of meta-analyses. Eur J Epidemiol 2020; 35:223-239.
2. Boccia, S., M. Hashibe, P. Gallì, E. De Feo, T. Asakage, T. Hashimoto, A. Hiraki, T. Katoh, T. Nomura, A. Yokoyama, C.M. van Duijn, G. Ricciardi, and P. Boffetta. Aldehyde dehydrogenase 2 and head and neck cancer: a meta-analysis implementing a Mendelian randomization approach. Cancer Epidemiol Biomarkers Prev 2009; 18:248-254.
3. Boes, E., S. Coassin, B. Kollerits, I.M. Heid, and F. Kronenberg. Genetic-epidemiological evidence on genes associated with HDL cholesterol levels: a systematic in-depth review. Exp Gerontol 2009; 44:136-160.
4. Budu-Aggrey, A., B. Brumpton, J. Tyrrell, S. Watkins, E.H. Modalsli, C. Celis-Morales, L.D. Ferguson, G. Vie, T. Palmer, L.G. Fritsche, M. Løset, J.B. Nielsen, W. Zhou, L.C. Tsoi, A.R. Wood, S.E. Jones, R. Beaumont, M. Saunes, P.R. Romundstad, S. Siebert, I.B. McInnes, J.T. Elder, G. Davey Smith, T.M. Frayling, B.O. Åsvold, S.J. Brown, N. Sattar, and L. Paternoster. Evidence of a causal relationship between body mass index and psoriasis: A mendelian randomization study. PLoS Med 2019; 16:e1002739.
5. Cao, A.L., Y.W. Lai, H.G. Chen, L.T. Sheng, and A. Pan. [Research progress of relationship between vitamin K and type 2 diabetes]. Zhonghua Yu Fang Yi Xue Za Zhi 2020; 54:555-562.
6. Chen, L., G. Davey Smith, R.M. Harbord, and S.J. Lewis. Alcohol intake and blood pressure: a systematic review implementing a Mendelian randomization approach. PLoS Med 2008; 5:e52.
7. Clarke, R., D.A. Bennett, S. Parish, P. Verhoef, M. Dötsch-Klerk, M. Lathrop, P. Xu, B.G. Nordestgaard, H. Holm, J.C. Hopewell, D. Saleheen, T. Tanaka, An, S.S. , J.C. Chambers, M.E. Kleber, Ouweh, W.H. , Y. Yamada, C. Elbers, B. Peters, A.F. Stewart, M.M. Reilly, Thor, B. , S. Yusuf, J.C. Engert, T.L. Assimes, J. Kooner, J. Danesh, H. Watkins, N.J. Samani, R. Collins, R. Peto, and M.S.C. Group. Homocysteine and coronary heart disease: meta-analysis of MTHFR case-control studies, avoiding publication bias. PLoS Med 2012; 9:e1001177.
8. Fluharty, M., A.E. Taylor, M. Grabski, and M.R. Munafò. The Association of Cigarette Smoking With Depression and Anxiety: A Systematic Review. Nicotine Tob Res 2017; 19:3-13.
9. Forouhi, N.G., Z. Ye, A.P. Rickard, K.T. Khaw, R. Luben, C. Langenberg, and N.J. Wareham. Circulating 25-hydroxyvitamin D concentration and the risk of type 2 diabetes: results from the European Prospective Investigation into Cancer (EPIC)-Norfolk cohort and updated meta-analysis of prospective studies. Diabetologia 2012; 55:2173-2182.
10. Fu, L., Y.N. Li, D. Luo, S. Deng, and Y.Q. Hu. Plausible relationship between homocysteine and obesity risk via MTHFR gene: a meta-analysis of 38,317 individuals implementing Mendelian randomization. Diabetes Metab Syndr Obes 2019; 12:1201-1212.
11. Fu, L., Y.N. Li, D. Luo, S. Deng, B. Wu, and Y.Q. Hu. Evidence on the causal link between homocysteine and hypertension from a meta-analysis of 40 173 individuals implementing Mendelian randomization. J Clin Hypertens (Greenwich) 2019; 21:1879-1894.
12. Harrison, S.C., A.J. Smith, G.T. Jones, D.I. Swerdlow, R. Rampuri, M.J. Bown, C. Aneurysm, L. Folkersen, A.F. Baas, G.J. de Borst, J.D. Blankensteijn, J.F. Price, Y. van der Graaf, S. McLachlan, O. Agu, A. Hofman, A.G. Uitterlinden, A. Franco-Cereceda, Y.M. Ruigrok, F.N. van't Hof, J.T. Powell, A.M. van Rij, J.P. Casas, P. Eriksson, M.V. Holmes, F.W. Asselbergs, A.D. Hingorani, and S.E. Humphries. Interleukin-6 receptor pathways in abdominal aortic aneurysm. Eur Heart J 2013; 34:3707-3716.
13. Hartwig, F.P., B.L. Horta, G.D. Smith, C.L. De Mola, and C.G. Victora. Association of lactase persistence genotype with milk consumption, obesity and blood pressure: A Mendelian randomization study in the 1982 Pelotas (Brazil) Birth Cohort, with a systematic review and meta-analysis. Int J Epidemiol 2016; 45:1573-1587.
14. He, L., B. Wang, W.Y. Lang, J. Xue, D.L. Zhao, G.F. Li, L.H. Zheng, and H.M. Pan. Genetically-reduced serum ACE activity might be a causal risk factor for obstructive sleep apnea syndrome: A meta-analysis. Sci Rep 2015; 5:15267.
15. Heath, A.K., I.Y. Kim, A.M. Hodge, D.R. English, and D.C. Muller. Vitamin D Status and Mortality: A Systematic Review of Observational Studies. Int J Environ Res Public Health 2019; 16(3):383.
16. Hu, Q., W. Teng, J. Li, F. Hao, and N. Wang. Homocysteine and Alzheimer's Disease: Evidence for a Causal Link from Mendelian Randomization. J Alzheimers Dis 2016; 52:747-756.
17. Huang, Q., J. Mi, X. Wang, F. Liu, D. Wang, D. Yan, B. Wang, S. Zhang, and G. Tian. 2016. Genetically lowered concentrations of circulating sRAGE might cause an increased risk of cancer: Meta-analysis using Mendelian randomization. J Int Med Res 2016; 44:179-191.
18. Jacobs, B.M., T. Taylor, A. Awad, D. Baker, G. Giovanonni, A. Noyce, and R. Dobson. 2020. Summary-data-based mendelian randomisation reveals druggable targets for multiple sclerosis. bioRxiv 2020.01.20.907451; doi: https://doi.org/10.1101/2020.01.20.907451, preprint: not peer reviewed.
19. Jacobs, B.M., T. Taylor, A. Awad, D. Baker, G. Giovanonni, A.J. Noyce, and R. Dobson. Summary-data-based Mendelian randomization prioritizes potential druggable targets for multiple sclerosis. Brain Commun 2020; 14;2(2):fcaa119.
20. Jiang, Y., Z. Su, C. Li, R. Wang, Y. Wen, H. Liang, J. He, and W. Liang. Association between the use of aspirin and risk of lung cancer: results from pooled cohorts and Mendelian randomization analyses. J Cancer Res Clin Oncol 2021; 147:139-151.
21. Khankari NK, Shu XO, Wen W, Kraft P, Lindström S, Peters U, Schildkraut J, Schumacher F, Bofetta P, Risch A, Bickeböller H, Amos CI, Easton D, Eeles RA, Gruber SB, Haiman CA, Hunter DJ, Chanock SJ, Pierce BL, Zheng W; Colorectal Transdisciplinary Study (CORECT); Discovery, Biology, and Risk of Inherited Variants in Breast Cancer (DRIVE); Elucidating Loci Involved in Prostate Cancer Susceptibility (ELLIPSE); Transdisciplinary Research in Cancer of the Lung (TRICL). Association between Adult Height and Risk of Colorectal, Lung, and Prostate Cancer: Results from Meta-analyses of Prospective Studies and Mendelian Randomization Analyses. PLoS Med. 2016;13(9):e1002118.
22. Kuper, H., A. Nicholson, M. Kivimaki, A. Aitsi-Selmi, G. Cavalleri, J.E. Deanfield, P. Heuschmann, X. Jouven, S. Malyutina, B.M. Mayosi, S. Sans, T. Thomsen, J.C.M. Witteman, A.D. Hingorani, D.A. Lawlor, and H. Hemingway. Evaluating the causal relevance of diverse risk markers: horizontal systematic review. BMJ: British Medical Journal (Overseas & Retired Doctors Edition) 2009; 339:4265-4265.
23. Lai, A.G., W.H. Chang, C.A. Parisinos, M. Katsoulis, R.M. Blackburn, A.D. Shah, V. Nguyen, S. Denaxas, G.D. Smith, T.R. Gaunt, K. Nirantharakumar, M.P. Cox, D. Forde, F. Asselbergs, S. Harris, S. Richardson, R. Sofat, R.J.B. Dobson, A. Hingorani, R. Patel, J. Sterne, A. Banerjee, A.K. Denniston, S. Ball, N.J. Sebire, N.H. Shah, G.R. Foster, B. Williams, and H. Hemingway. 2021. An Informatics Consult approach for generating clinical evidence for treatment decisions. medRxiv 2021.01.10.21249331; doi: <https://doi.org/10.1101/2021.01.10.21249331>, preprint: not peer reviewed.
24. Lewis, S.J., I. Baker, and G.D. Smith. Meta-analysis of vitamin D receptor polymorphisms and pulmonary tuberculosis risk. Int J Tuberc Lung Dis. 9:1174-1177.
25. Li, M., E. Francis, S.N. Hinkle, A.S. Ajjarapu, and C. Zhang. 2019. Preconception and Prenatal Nutrition and Neurodevelopmental Disorders: A Systematic Review and Meta-Analysis. Nutrients 2005; 11:1628-1628.
26. Liu, Z., S. Liang, S. Que, L. Zhou, S. Zheng, and A. Mardinoglu. Meta-Analysis of Adiponectin as a Biomarker for the Detection of Metabolic Syndrome. Front Physiol 2018; 9:1238.
27. Lupo, P.J., L.E. Mitchell, and M.M. Jenkins. Genome-wide association studies of structural birth defects: A review and commentary. Birth Defects Res 2019; 111:1329-1342.
28. Marshe, V.S., I. Gorbovskaya, S. Kanji, M. Kish, and D.J. Muller. 2019. Clinical implications of APOE genotyping for late-onset Alzheimer's disease (LOAD) risk estimation: a review of the literature. J Neural Transm (Vienna) 2019; 126:65-85.
29. Matejcic, M., M.J. Gunter, and P. Ferrari. Alcohol metabolism and oesophageal cancer: A systematic review of the evidence. Carcinogenesis 2017; 38:859-872.
30. Niu, W., Q. Pang, T. Lin, Z. Wang, J. Zhang, M. Tai, L. Zhang, L. Zhang, M. Gu, C. Liu, and K. Qu. A Causal Role of Genetically Elevated Circulating Interleukin-10 in the Development of Digestive Cancers: Evidence from Mendelian Randomization Analysis Based on 29,307 Subjects. Medicine 2016; 95:e2799.
31. Niu, W., and Y. Qi. Circulating cholesteryl ester transfer protein and coronary heart disease: Mendelian randomization meta-analysis. Circ Cardiovasc Genet 2015; 8:114-121.
32. Palmer, T.M., J.R. Thompson, and M.D. Tobin. Meta-analysis of Mendelian randomization studies incorporating all three genotypes. Stat Med 2008; 27:6570-6582.
33. Pei, Y., Y. Xu, and W. Niu. Causal relevance of circulating adiponectin with cancer: a meta-analysis implementing Mendelian randomization. Tumor Biol 2015; 36:585-594.
34. Peinemann, F., C. Bartel, and U. Grouven. First-line allogeneic hematopoietic stem cell transplantation of HLA-matched sibling donors compared with first-line ciclosporin and/or antithymocyte or antilymphocyte globulin for acquired severe aplastic anemia. Cochrane Database Syst Rev 2013; CD006407.
35. Peinemann, F., and A.M. Labeit. Stem cell transplantation of matched sibling donors compared with immunosuppressive therapy for acquired severe aplastic anaemia: A Cochrane systematic review. BMJ Open 2014; 4:e005039.
36. Peng, H., C. Li, X. Wu, Y. Wen, J. Lin, H. Liang, R. Zhong, J. Liu, J. He, W. Liang, H. Peng, X. Wu, Y. Wen, J. Lin, J. He, and W. Liang. 2020. Association between systemic lupus erythematosus and lung cancer: Results from a pool of cohort studies and Mendelian randomization analysis. J. Thorac. Dis 2020; 12:5299-5312.
37. Peng, H., X. Wu, Y. Wen, C. Li, J. Lin, J. Li, S. Xiong, R. Zhong, H. Liang, B. Cheng, J. Liu, J. He, and W. Liang. Association between systemic sclerosis and risk of lung Cancer: Results from a Pool of cohort studies and Mendelian randomization analysis. Autoimmun Rev 2020; 19:102633.
38. Policicchio, S., A.N. Ahmad, J.F. Powell, and P. Proitsi. Rheumatoid arthritis and risk for Alzheimer's disease: a systematic review and meta-analysis and a Mendelian Randomization study. Sci Rep 2017; 7:12861.
39. Rice, F., K. Langley, C. Woodford, G. Davey Smith, and A. Thapar. Identifying the contribution of prenatal risk factors to offspring development and psychopathology: What designs to use and a critique of literature on maternal smoking and stress in pregnancy. Dev Psychopathol 2018; 30:1107-1128.
40. Schmidt, A.F., N.B. Hunt, M. Gordillo-Maranon, P. Charoen, F. Drenos, J.-P. Casas, M. Kivimaki, D.A. Lawlor, C. Giambartolomei, O. Papacosta, N. Chaturvedi, J.C. Bis, #039, C. Donnell, G. Wannamethee, A. Wong, J.F. Price, A.D. Hughes, T.R. Gaunt, N. Franceschini, D.O. Mook-Kanamori, M. Zwierzyna, R. Sofat, A.D. Hingorani, and C. Finan. 2020. Cholesteryl Ester Transfer Protein as a Drug Target for Cardiovascular Disease. medRxiv 2020.09.07.20189571; doi: https://doi.org/10.1101/2020.09.07.20189571 preprint: not peer reviewed.
41. Song, C., S. Burgess, J.D. Eicher, C.J. O'Donnell, and A.D. Johnson. Causal Effect of Plasminogen Activator Inhibitor Type 1 on Coronary Heart Disease. J Am Heart Assoc 2017; 26;6(6):e004918
42. Syed, A.A.S., L. He, Y. Shi, and S. Mahmood. Elevated levels of IL-18 associated with schizophrenia and first episode psychosis: A systematic review and meta-analysis. Early Interv Psychiatry 2020; 15(4):896-905
43. Yu, X., Z. Yuan, H. Chen, J. Yang, Y. Gao, F. Guan, P. Zeng, and S. Huang. 2019. Relationship between birth weight and chronic kidney disease: an integrative analysis of observational studies and causal inference through genetic approaches. bioRxiv 729715; doi: <https://doi.org/10.1101/729715>, preprint: not peer reviewed.
44. Yu, X., Z. Yuan, H. Lu, Y. Gao, H. Chen, Z. Shao, J. Yang, F. Guan, S. Huang, and P. Zeng. Relationship between birth weight and chronic kidney disease: Evidence from systematics review and two-sample Mendelian randomization analysis. Hum Mol Genet 2020; 29:2261-2274.
45. Yuan, S., Y. Xiong, and S.C. Larsson. An atlas on risk factors for multiple sclerosis: a Mendelian randomization study. J Neurol 2021; 268:114-124.
46. Zeng, Z., W. Zhang, Y. Qian, H. Huang, D.J.H. Wu, Z. He, D. Ye, Y. Mao, and C. Wen. Association of telomere length with risk of rheumatoid arthritis: a meta-analysis and Mendelian randomization. Rheumatology (Oxford) 2020; 59:940-947.

**Protocols of systematic reviews of Mendelian randomization studies (search 3)**

Do not include MR studies (15 references):

1. Cara, K.C., A.R. Beauchesne, M. Chung, and T.C. Wallace. A Systematic Review and Research Gap Analysis on the Effects of 100% Orange Juice on Oxidative Stress and Inflammation Markers. PROSPERO 2021 CRD42021235438 Available from: https://www.crd.york.ac.uk/prospero/display_record.php?ID=CRD42021235438.
2. Chen, Z., S. Liang, and Y. Bai. Association between serum uric acid and major depressive disorder: a meta-analysis and two-sample bidirectional mendelian randomisation. PROSPERO 2020 CRD42020199931 Available from: https://www.crd.york.ac.uk/prospero/display_record.php?ID=CRD42020199931.
3. Friel, C., R. Dundas, A. Leyland, J. Anderson, M. Shimonovich, T. Borge, and A. Havdahl. Nutritional status in pregnancy and Autism Spectrum Disorder in offspring: a social perspective [systematic review and meta-analysis]. PROSPERO 2019 CRD42019154613 Available from: https://www.crd.york.ac.uk/prospero/display_record.php?ID=CRD42019154613.
4. Giannandrea, D., and F. Paciullo. Lipoprotein (a) and risk of retinal vein occlusion: a review and meta-analysis. PROSPERO 2020 CRD42020196552 Available from: https://www.crd.york.ac.uk/prospero/display_record.php?ID=CRD42020196552.
5. Gu, D., and B. Zhang. Association between systemic lupus erythematosus and cancer: findings from cohort studies and Mendelian randomization analysis. PROSPERO 2021 CRD42021243635 Available from: https://www.crd.york.ac.uk/prospero/display_record.php?ID=CRD42021243635.
6. Guijosa, A., A. Armenta Bani, A. Aguirre, J.C. Cruz Lopez, and A.C. Perez-Ortiz. Pharmacogenetics of Taxane-Induced Neurotoxicity in Breast Cancer: Systematic Review and Meta-Analysis. PROSPERO 2020 CRD42020197199 Available from:

<https://www.crd.york.ac.uk/prospero/display_record.php?ID=CRD42020197199>.

1. Xie J., Liu, W.. Association between childhood asthma and type 1 diabetes: results

from a pool of cohort studies and Mendelian randomization analysis. PROSPERO 2021

CRD42021236232 Available from:

https://www.crd.york.ac.uk/prospero/display_record.php?ID=CRD42021236232.

1. Lassale, C., and M.T. Soria Florido. HDL-cholesterol functionality and CVD outcomes: a systematic review and meta-analysis. PROSPERO 2017 CRD42017065857 Available from:

https://www.crd.york.ac.uk/prospero/display_record.php?ID=CRD42017065857.

1. Mao, Y., L. Lv, and J. Chen. Dietary phosphorus intakes and the risk of prostate cancer: a systematic review and meta-analysis. . PROSPERO 2021 CRD42021232964 Available from: https://www.crd.york.ac.uk/prospero/display_record.php?ID=CRD42021232964.
2. Mao, Y., W. Zhang, Z. Zeng, and D. Ye. Association of telomere length with risk of rheumatoid arthritis: a meta-analysis and Mendelian randomization. PROSPERO 2019 CRD42019136646 Available from: https://www.crd.york.ac.uk/prospero/display_record.php?ID=CRD42019136646.
3. Najjar, L., E. Hypponen, J. Sutherland, and A. Zhou. Vitamin D and type 1 diabetes risk: A systematic review and meta-analysis of genetic evidence to help establish a causal association. PROSPERO 2021 CRD42021224844 Available from: https://www.crd.york.ac.uk/prospero/display_record.php?ID=CRD42021224844.
4. Roberts, S., D. Vears, J. Minion, and M. Murtagh. Stakeholder views on the return of results from genomic research: a systematic review of quantitative and qualitative studies. PROSPERO 2018 CRD42018117551 Available from: https://www.crd.york.ac.uk/prospero/display_record.php?ID=CRD42018117551.
5. Singh, T.P., M. Field, M. Bown, G. Jones, and J. Golledge. Systematic review of genome wide association studies of abdominal aortic aneurysm. PROSPERO 2021 CRD42021229826 Available from: <https://www.crd.york.ac.uk/prospero/display_record.php?ID=CRD42021229826>.
6. Tsartsalis, D., D. Korela, A. Anastasakis, E. Foukarakis, D. Venetsanos, E. Dragioti, and E. Charitakis. Risk and protective factors for sudden cardiac death: an umbrella review of meta-analyses. PROSPERO 2020 CRD42020216363 Available from: https://www.crd.york.ac.uk/prospero/display_record.php?ID=CRD42020216363.
7. Widding-Havneraas, T.C., A., I. Lyhmann, H.D. Zachrisson, F. Elwert, D. McDaid, and A. Mykletun. Applications of preference-based instrumental variables in health research: a systematic review. PROSPERO 2020 CRD42020165014 Available from:

https://www.crd.york.ac.uk/prospero/display_record.php?ID=CRD42020165014.

**5. Supplementary Table S1: List of included studies containing one or more tools for evaluating, conducting, and reporting Mendelian randomization (MR) studies**

| **Study ID** | **Type of article** | **Scope of the tool(s)** | **Number of tools** | **Structure of the tool** |
| --- | --- | --- | --- | --- |
| **Boef 2015^a1^** | Systematic review of methodological approaches and  quality of reporting in MR studies | Reporting | 1 | Checklist |
| **Burgess 2020^2^** | Guideline for performing MR investigations | Assessing  Conducting  Reporting | 2 | Domain-based checklist  (assessing and reporting tool)  Flowchart (conducting tool) |
| **Davey Smith 2019^3^** | STROBE-MR; guideline for the reporting of MR studies | Reporting | 1 | Checklist |
| **Davies 2018^4^** | Guide, checklist and glossary for MR studies | Assessing  Reporting | 1 | Checklist |
| **Grau-Perez 2019^a5^** | Systematic review of MR studies | Assessing  Reporting | 1 | Domain-based evaluation chart |
| **Grover 2015^6^** | Guideline for performing MR analysis | Conducting | 1 | Flowchart |
| **Kuźma 2018^a7^** | Systematic review of MR studies | Assessing  Reporting | 1 | Rating scale |
| **Lawlor 2019^8^** | MR dictionary and online tool | Conducting | 1 | Flowchart |
| **Lee 2020^b9^** | Protocol for a systematic review of MR studies on risk factors for dementia | Assessing  Reporting | 1 | Questionnaire |
| **Lor 2019^a10^** | Systematic review of MR studies and guideline for reporting MR studies | Reporting | 1 | Checklist |
| **Mamluk 2020^a11^** | Systematic review of MR studies | Assessing | 1 | Bias domain-based rating |
| **Swerdlow 2016^a12^** | Review of selection of instrument in MR analysis and systematic review of MR studies | Conducting | 1 | Decision tree |
| **Treur 2021^a13^** | Systematic review of MR studies | Assessing  Reporting | 1 | Scoring system |

^a^ Included in the synthesis of systematic review of MR studies. ^b^ Included in the synthesis of protocols of systematic reviews of MR studies. Abbreviations: MR=Mendelian randomization.

**6. Supplementary Table S2: Details of other Mendelian randomization (MR)-relevant content of items or questions within each assessing tool**

| **Aspect of MR analysis** | **Specific topic** | **Burgess 2020** | **Davies 2018** | **Grau-Perez 2019** | **Kuzma 2018** | **Lee 2020** | **Mamluk 2020** | **Treur 2021** | **Total** |
| --- | --- | --- | --- | --- | --- | --- | --- | --- | --- |
| **Clinical implications** | Reporting of clinical implication |  | Y |  |  |  |  |  | 2 |
| **Datasets** | Reporting of datasets used |  | Y |  |  | Y |  |  | 2 |
| **Genetic instrument** | Reporting of MR rationale (biological rationale) | Y |  |  |  |  |  |  | 1 |
|  | Reporting of method used to obtain genetic variant |  |  | Y |  |  |  |  | 1 |
|  | Reporting of genetic variant strength |  |  |  |  |  |  | Y | 2 |
| **Interpretation** | Applicability/transportability |  | Y |  |  |  |  |  | 1 |
|  | Interpretation of results |  | Y |  |  |  |  |  | 2 |
|  | Reporting of MR estimates for interpretation of results |  | Y |  |  |  |  |  | 1 |
|  | Bidirectional effects |  |  |  |  |  |  | Y | 1 |
|  | Temporality |  |  |  |  |  |  | Y | 1 |
| **MR rationale** | Reporting of MR rationale | Y |  |  | Y |  |  | Y | 5 |
| **MR results** | Reporting of comparison of MR estimate with observational study estimate |  | Y | Y |  | Y |  |  | 3 |
|  | Reporting of MR analysis results |  | Y | Y |  |  |  |  | 3 |
| **Precision** | Statistical power/sample size |  |  | Y | Y |  |  | Y | 4 |
| **Selection of population/sample** | Gathering specific methods details | Y |  |  |  |  |  |  | 1 |
|  | Reporting of relevance of the selected population to the research question | Y |  |  |  |  |  |  | 1 |
| **Statistical analysis** | Reporting of statistical methods |  |  | Y | Y |  |  |  | 2 |
|  | Reporting of MR analysis results | Y |  |  |  |  |  |  | 1 |
|  | Reporting of primary analysis statistical methods | Y |  |  |  |  |  |  | 1 |
|  | Reporting of primary analysis details | Y |  |  |  |  |  | Y | 2 |
|  | Reporting of secondary analysis statistical methods | Y |  |  |  |  |  |  | 1 |
| **Type of dataset** | Gathering specific methods details | Y |  |  |  |  |  |  | 1 |

Abbreviations: MR=Mendelian randomization; Y=yes.

**7. Supplementary Table S3: Details of reporting and conducting tools**

| **Author** | **Objectives of the tool** | **Scope of the tool** | **Tool used as template /reference to other tools or articles relevant to MR** | **n of domains** | **n of items** | **Details of bias addressed** |
| --- | --- | --- | --- | --- | --- | --- |
| **Boef 2015** | To provide an overview of the use of the different approaches to MR and the specific statistical methods used for estimation. To evaluate whether the plausibility of the MR assumptions is discussed. To evaluate whether the statistical methods used are sufficiently described. | Reporting | NR | 3 | 7 | Weak instrument; confounders of GI-exposure association; unmeasured confounders of GI-outcome association; population stratification; horizontal pleiotropy; linkage disequilibrium, canalization; |
| **Davey Smith 2019(STROBE-MR)** | To provide guidelines for strengthening the reporting of MR studies. | Reporting | STROBE ^14^ | 6 | 44 | IV1-3; homogeneity; sample overlap; |
| **Lor 2019** | To evaluate the reporting quality of MR analyses in cancer studies and provide guidelines for reporting in MR biomedical research, for use in future publications. | Reporting | PRISMA ^15^  STREGA ^16^  STROBE ^14^ | 6 | 44^a^; 61^b^ | Weak instrument; confounders of GI-exposure and/or GI-outcome association; population stratification; horizontal pleiotropy; linkage disequilibrium; heteroscedasticity; |
| **Burgess 2020** | To provide guidelines for performing MR investigations. To provide advice on which analyses to perform in a MR investigation. | Conducting | NR | 9 | 26 | IV1-3 assumptions; variants harmonization; homogeneity assumption; |
| **Grover 2015** | To provide a step-by-step guide for causal inference based on the principles of MR with a real dataset using both individual and summary data from unrelated individuals. | Conducting | NR | 6 | 24 | Sample overlap; variants harmonization; confounders of GI-exposure and/or GI-outcome association; strength of genetic variant; horizontal pleiotropy; |
| **Lawlor 2019** | To provide definitions and key references for terms and concepts that are necessary to undertake MR studies, and to critically appraise and appropriately interpret findings from MR studies. | Conducting | NR | 10 | 18 | Sample overlap; variants harmonization; confounders of GI-exposure and/or GI-outcome association; strength of genetic variant; horizontal pleiotropy; |
| **Swerdlow 2016** | To provide guidance that may help investigators to plan a Mendelian randomization study of a disease-associated biomarker, and readers interpret Mendelian randomization studies. | Conducting | NR | 5 | 20 | Linkage disequilibrium; horizontal pleiotropy; |

Abbreviations: GI=genetic instrument; IV=instrumental variable; MR=Mendelian randomization; NR=not reported; PRISMA=Preferred Reporting Items for Systematic Reviews and Meta-Analyses; STREGA= STrengthening the REporting of Genetic Association studies; STROBE=Strengthening the Reporting of Observational Studies in Epidemiology. ^a^Studies with data pooling; ^b^Studies without data pooling.

**8. Supplementary Table S4: Details of bias addressed by a narrative description in systematic reviews of Mendelian randomization (MR) studies.**

|  | **IV1 (Relevance)** | **IV2**  **(Independence)** | | | | | **IV3**  **(Exclusion restriction)** |
| --- | --- | --- | --- | --- | --- | --- | --- |
| **Study ID** | **Weak instrument** | **Confounding** | **Population stratification** | **Assortative mating** | **Dynastic effect** | **Parent of origin effect** | **Horizontal pleiotropy** |
| **Abbasi 2015** |  | Y |  |  |  |  | Y |
| **Abbasi 2016** |  |  |  |  |  |  | Y |
| **Belbasi 2018** | Y | Y |  |  |  |  | Y |
| **Bellou 2018** | Y |  |  |  |  |  |  |
| **Bergmans 2021** |  |  |  |  |  |  | Y |
| **Bochud 2010** | Y | Y | Y |  |  | Y | Y |
| **Boef 2015** | Y | Y | Y |  |  |  | Y |
| **Carnegie 2020** | Y |  | Y |  |  |  | Y |
| **Diemer 2021** | Y | Y | Y | Y |  |  | Y |
| **Firth 2020** |  |  |  |  |  |  | Y |
| **Frayling 2018** | Y | Y | Y |  |  |  | Y |
| **Grau-Perez 2019** | Y | Y | Y |  |  |  | Y |
| **Hu 2019** | Y |  | Y |  |  |  | Y |
| **Kei 2018** | Y |  |  |  |  |  | Y |
| **Kim 2020** | Y |  |  |  |  |  |  |
| **Kohler 2018** |  |  |  |  |  |  |  |
| **Kuzma 2018** | Y |  | Y |  |  |  | Y |
| **Li 2017** | Y | Y |  |  |  |  | Y |
| **Lor 2019** | Y | Y^a^ | Y | Y |  |  | Y |
| **Mamluk 2020** | Y | Y | Y | Y | Y |  | Y |
| **Markozannes 2021** |  |  |  |  |  |  | Y |
| **Pearson-Stuttard 2021** |  |  |  |  |  |  | Y |
| **Pingault 2016** | Y |  | Y |  |  |  | Y |
| **Riaz 2018** | Y |  |  |  |  |  | Y |
| **Robinson 2016** | Y | Y | Y |  |  |  | Y |
| **Swerdlow 2016** | Y |  |  |  |  |  | Y |
| **Treur 2121** |  | Y | Y | Y | Y |  | Y |
| **Zhang 2019** | Y |  |  |  |  |  |  |

**Supplementary Table S4 (continued)**

|  | **Selection of genetic instrument** | | | | | **Selection of sample** | |
| --- | --- | --- | --- | --- | --- | --- | --- |
| **Study ID** | **Linkage disequilibrium** | **Winner's curse^b^** | **Segregation distortion** | **Lack of monotonicity** | **Allelic homogeneity** | **Population heterogeneity** | **Selection bias** |
| **Abbasi 2015** |  |  |  |  |  |  |  |
| **Abbasi 2016** |  |  |  |  |  |  |  |
| **Belbasi 2018** |  |  |  |  |  |  |  |
| **Bellou 2018** |  |  |  |  |  |  |  |
| **Bergmans 2021** |  |  |  |  |  |  |  |
| **Bochud 2010** | Y |  | Y |  | Y |  | Y^c^ |
| **Boef 2015** |  | Y |  |  |  |  |  |
| **Carnegie 2020** | Y |  |  |  |  |  |  |
| **Diemer 2021** |  | Y |  |  |  | Y | Y |
| **Firth 2020** |  |  |  |  |  |  |  |
| **Frayling 2018** |  |  |  |  |  |  |  |
| **Grau-Perez 2019** | Y |  |  |  |  |  |  |
| **Hu 2019** | Y |  |  |  |  |  |  |
| **Kei 2018** | Y |  |  |  |  |  |  |
| **Kim 2020** |  |  |  |  |  |  |  |
| **Kohler 2018** |  |  |  |  |  |  |  |
| **Kuzma 2018** |  |  |  |  |  | Y | Y |
| **Li 2017** |  |  |  |  |  |  |  |
| **Lor 2019** | Y |  |  |  |  | Y |  |
| **Mamluk 2020** |  |  |  |  |  |  | Y |
| **Markozannes 2021** |  |  |  |  |  |  |  |
| **Pearson-Stuttard 2021** |  |  |  | Y |  |  |  |
| **Pingault 2016** | Y |  |  |  |  |  |  |
| **Riaz 2018** |  |  |  |  |  |  |  |
| **Robinson 2016** | Y |  |  |  |  |  |  |
| **Swerdlow 2016** | Y | Y |  |  |  |  |  |
| **Treur 2121** | Y |  |  |  |  |  | Y |
| **Zhang 2019** | Y |  |  |  |  |  |  |

**Supplementary Table S4 (continued)**

|  | **Canalization** | **Measurement errors / misclassification** | **Lack of genetic instrument** | **Reverse causation (or bidirectionality)** | **Inability to assess non-linear associations** | **Inability to assess dose–response estimation** | **Low statistical power** | **Statistical analysis** |
| --- | --- | --- | --- | --- | --- | --- | --- | --- |
| **Abbasi 2015** | Y |  |  |  |  |  |  |  |
| **Abbasi 2016** | Y |  |  |  |  |  |  |  |
| **Belbasi 2018** |  |  |  |  |  |  |  |  |
| **Bellou 2018** |  |  |  |  |  |  | Y |  |
| **Bergmans 2021** |  |  |  |  |  |  |  |  |
| **Bochud 2010** | Y |  |  |  |  |  |  |  |
| **Boef 2015** |  |  |  |  |  |  |  |  |
| **Carnegie 2020** | Y |  | Y |  | Y |  | Y |  |
| **Diemer 2021** |  | Y |  |  |  |  | Y | Y |
| **Firth 2020** |  |  |  | Y | Y | Y | Y |  |
| **Frayling 2018** |  |  |  |  |  |  |  |  |
| **Grau-Perez 2019** |  |  |  |  |  |  |  |  |
| **Hu 2019** | Y |  |  |  |  |  | Y |  |
| **Kei 2018** | Y |  |  |  |  |  |  |  |
| **Kim 2020** |  |  |  | Y |  |  | Y |  |
| **Kohler 2018** |  |  |  |  |  |  | Y |  |
| **Kuzma 2018** |  |  |  |  |  |  |  |  |
| **Li 2017** |  |  |  |  |  |  |  |  |
| **Lor 2019** |  |  |  |  |  |  | Y |  |
| **Mamluk 2020** | Y | Y |  | Y | Y |  | Y |  |
| **Markozannes 2021** |  |  |  |  |  |  |  |  |
| **Pearson-Stuttard 2021** |  | Y |  |  |  |  |  |  |
| **Pingault 2016** |  | Y |  | Y |  |  | Y |  |
| **Riaz 2018** |  |  |  |  |  |  |  |  |
| **Robinson 2016** | Y |  |  |  |  |  | Y |  |
| **Swerdlow 2016** |  |  |  |  |  |  |  |  |
| **Treur 2121** |  |  |  | Y |  |  |  |  |
| **Zhang 2019** |  |  |  |  |  |  |  | Y |

^a^ Confounding by shared genetics and environment. ^b^ In selecting genetic instruments; ^c^ Selective survival due to the genetic variant of interest. Abbreviations: IV1= instrumental variable assumption 1; IV2= instrumental variable assumption 2; IV3= instrumental variable assumption 3; Y=yes.

**References**

1. Boef AGC, Dekkers OM, Le Cessie S. Mendelian randomization studies: A review of the approaches used and the quality of reporting. International Journal of Epidemiology 2015; 44(2): 496-511.

2. Burgess S, Davey Smith G, Davies NM, et al. Guidelines for performing Mendelian randomization investigations [version 2; peer review: 2 approved]. Wellcome Open Research 2020; 4(186).

3. Davey Smith G, Davies NM, Dimou N, et al. STROBE-MR: Guidelines for strengthening the reporting of Mendelian randomization studies. PeerJ Preprints 2019; 7: e27857v1.

4. Davies NM, Holmes MV, Davey Smith G. Reading Mendelian randomisation studies: a guide, glossary, and checklist for clinicians. BMJ 2018; 362: k601.

5. Grau-Perez M, Agha G, Pang Y, Bermudez JD, Tellez-Plaza M. Mendelian Randomization and the Environmental Epigenetics of Health: a Systematic Review. Current environmental health reports 2019; 6(1): 38-51.

6. Grover S, Del Greco M. F, Stein CM, Ziegler A. Mendelian Randomization. In: Elston RC, ed. Statistical Human Genetics: Methods and Protocols. New York, NY: Springer New York; 2017: 581-628.

7. Kuźma E, Hannon E, Zhou A, et al. Which Risk Factors Causally Influence Dementia? A Systematic Review of Mendelian Randomization Studies. Journal of Alzheimer's disease : JAD 2018; 64(1): 181-93.

8. Lawlor DA, Wade K, Borges MC, et al. A Mendelian Randomization dictionary: Useful definitions and descriptions for undertaking, understanding and interpreting Mendelian Randomization studies. OSF Preprints 2019.

9. Lee LS, Whiteley W, Walker R. Systematic review and meta-analysis of Mendelian randomisation studies on modifiable risk factors for dementia. protocols.io

https://dx.doi.org/10.17504/protocols.io.bpeemjbe.

10. Lor GCY, Risch HA, Fung WT, et al. Reporting and guidelines for mendelian randomization analysis: A systematic review of oncological studies. Cancer Epidemiology 2019; 62: 101577.

11. Mamluk L, Jones T, Ijaz S, et al. Evidence of detrimental effects of prenatal alcohol exposure on offspring birthweight and neurodevelopment from a systematic review of quasi-experimental studies. Int J Epidemiol 2021; 49(6): 1972-95.

12. Swerdlow DI, Kuchenbaecker KB, Shah S, et al. Selecting instruments for Mendelian randomization in the wake of genome-wide association studies. International Journal of Epidemiology 2016; 45(5): 1600-16.

13. Treur JL, Munafo MR, Logtenberg E, Wiers RW, Verweij KJH. Using Mendelian randomization analysis to better understand the relationship between mental health and substance use: a systematic review. Psychol Med 2021: 1-32.

14. von Elm E, Altman DG, Egger M, et al. The Strengthening the Reporting of Observational Studies in Epidemiology (STROBE) statement: guidelines for reporting observational studies. Lancet 2007; 370(9596): 1453-7.

15. Moher D, Liberati A, Tetzlaff J, Altman DG, Group P. Preferred reporting items for systematic reviews and meta-analyses: the PRISMA statement. PLoS Med 2009; 6(7): e1000097.

16. Little J, Higgins JP, Ioannidis JP, et al. STrengthening the REporting of Genetic Association studies (STREGA)--an extension of the STROBE statement. Eur J Clin Invest 2009; 39(4): 247-66.
